# Strategies to Improve the Potential Functionality of Fruit-Based Fermented Beverages

**DOI:** 10.3390/plants10112263

**Published:** 2021-10-22

**Authors:** Ancuța-Liliana Keșa, Carmen Rodica Pop, Elena Mudura, Liana Claudia Salanță, Antonella Pasqualone, Cosmin Dărab, Cristina Burja-Udrea, Haifeng Zhao, Teodora Emilia Coldea

**Affiliations:** 1 Department of Food Engineering, Faculty of Food Science and Technology, University of Agricultural Sciences and Veterinary Medicine Cluj-Napoca, 400372 Cluj-Napoca, Romania; kesaancuta@yahoo.com (A.-L.K.); elena.mudura@usamvcluj.ro (E.M.); 2 Department of Food Science, Faculty of Food Science and Technology, University of Agricultural Sciences and Veterinary Medicine, 400372 Cluj-Napoca, Romania; carmen-rodica.pop@usamvcluj.ro (C.R.P.); liana.salanta@usamvcluj.ro (L.C.S.); 3 Department of Soil, Plant and Food Sciences, University of Bari ‘Aldo Moro’, Via Amendola, 165/A, 70126 Bari, Italy; antonella.pasqualone@uniba.it; 4 Department of Electric Power Systems, Faculty of Electrical Engineering, Technical University of Cluj-Napoca, 400027 Cluj-Napoca, Romania; cosmin.darab@enm.utcluj.ro; 5 Industrial Engineering and Management Department, Faculty of Engineering, Lucian Blaga University of Sibiu, 10 Victoriei Blv., 550024 Sibiu, Romania; cristina.udrea@ulbsibiu.ro; 6 School of Food Science and Engineering, South China University of Technology, Guangzhou 510640, China; hfzhao@scut.edu.cn; 7 Research Institute for Food Nutrition and Human Health, Guangzhou 510640, China

**Keywords:** fermentation, functional, fermented beverages, bioactive compounds

## Abstract

It is only recently that fermentation has been facing a dynamic revival in the food industry. Fermented fruit-based beverages are among the most ancient products consumed worldwide, while in recent years special research attention has been granted to assess their functionality. This review highlights the functional potential of alcoholic and non-alcoholic fermented fruit beverages in terms of chemical and nutritional profiles that impact on human health, considering the natural occurrence and enrichment of fermented fruit-based beverages in phenolic compounds, vitamins and minerals, and pro/prebiotics. The health benefits of fruit-based beverages that resulted from lactic, acetic, alcoholic, or symbiotic fermentation and specific daily recommended doses of each claimed bioactive compound were also highlighted. The latest trends on pre-fermentative methods used to optimize the extraction of bioactive compounds (maceration, decoction, and extraction assisted by supercritical fluids, microwave, ultrasound, pulsed electric fields, high pressure homogenization, or enzymes) are critically assessed. As such, optimized fermentation processes and post-fermentative operations, reviewed in an industrial scale-up, can prolong the shelf life and the quality of fermented fruit beverages.

## 1. Introduction

Fruit-based beverages are among the oldest and most traditional fermented products. Fruits are characterized by high sugar content and thus are a raw material particularly suitable for alcoholic fermentation and other fermentations, such as acetic or lactic fermentation. Numerous examples can be mentioned for both alcoholic and non-alcoholic fermented fruit-based beverages, such as vinegar, fruit beer, water kefir [1], cider, wine (both conventional wine and wines from fruits other than grape), and several less common traditional products such as the Turkish *hardaliye*, the Namibian *ombike* and *omalunga*, the Mexican *tepache*, the Rwandese *urwagwa*, the *setopoti* and the *khadi* from Botswana, all described in detail in Section 1.1.

Interest in the consumption of fermented beverages has been steadily increasing due to consumer awareness of their positive effect on human health, mostly related to the content of bioactive compounds and their purported probiotic effect. Several methods have been therefore proposed to improve the extraction of bioactive compounds from the raw materials, both at the pre-fermentative stage (by simple maceration, decoction, and by extraction assisted by supercritical fluids, microwave, ultrasound, or enzymes) and during fermentation or post-fermentation, using optimized processes and technologies that are able to prolong the shelf life and quality of fermented fruit beverages [2,3,4,5,6,7,8,9,10,11,12,13,14,15,16,17,18,19].

Recent work in the field has highlighted the microbial community as well as the health benefits of yeast and/or lactic acid bacteria in fermented fruit beverages and other food products [20,21,22,23,24,25], the technical limitations for their industrial upscaling [20], the quality of the ingredients and safety aspects related to traditional fermented foods and beverages [26,27], the new ways to analyze their fermentation processes [28], and the recent trends related to the consumption of fermented fruit-based beverages [29,30,31].

Nevertheless, many of the most widespread fruit-based fermented beverages are alcoholic, i.e., have an alcohol content higher than 1.2% (the lower limit according to EU rules), which must be indicated on their label. Moreover, it has to be considered that alcoholic beverages containing more than 1.2% alcohol by volume (ABV) cannot bear health claims on the label. As such, in order to take advantage of the potentially functional properties of fruit-based beverages, dealcoholization processes are therefore of paramount importance and have been extensively reviewed by Mangindaan et al. [32].

Assuming alcohol content reduction below 1.2% is technically feasible and has already been reviewed, the aim of this work is to highlight the functional potential of fermented fruit beverages in terms of chemical and nutritional profiles and to give an overview on the possible strategies to improve the potential functionality of these beverages. The latest trends on pre-fermentative methods used to optimize the extraction of bioactive compounds are critically assessed, as well as the optimized fermentation and post-fermentation operations, in view of an industrial scale-up.

### 1.1. Major and Minor Fruit-Based Fermented Beverages

With more than 3.2 million liters consumed every day in China due to its nutritional benefits [33], vinegar is available worldwide in several different assortments. Vinegar’s traditional use dates back to ancient times [34], even if there is no clear information about its origin [35]. Vinegar was defined by the joint commission of the FAO/WHO as “a liquid fit for human consumption, produced from a suitable raw material of agricultural origin, containing starch, sugars or starch and sugars, by the process of double fermentation, alcoholic and acetous, that can contain a specified amount of acetic acid” [36]. At present, wine vinegar is the most widely marketed, although vinegar can be produced from various raw materials [34]. If solely fruits are considered, it can be prepared from pineapple, including pineapple by-products [37,38], persimmon, apple, jujube [39], pomegranate [40], plum, cranberry [41], sour cherry [42], soursop [43], mango, orange, banana [44], and papaya [45]. Initially, juices are typically inoculated with *Saccharomyces cerevisiae* to allow alcoholic fermentation, converting sugars into ethanol under anaerobic conditions. The addition of *Acetobacter* species is the second stage of fermentation, enabling ethanol oxidation under aerobic conditions and its conversion into acetic acid [33]. The earliest methods used to produce vinegar include the Orléans method, generator method, and submerged culture method [45]. Vinegar is used primarily as an ingredient in food processing due to its taste and aroma [46,47], as a seasoning and preservative [41], as a marinating liquid for meat [48], or in sauces, ketchup, and mayonnaise [33], and recently as an ingredient for beverage formulations [44].

Similarly, beer, which is the most consumed alcoholic beverage worldwide [49], is produced by the saccharification of starch and fermentation of the resulting glucose, maltose, and maltotriose [50]. Fruit beers, primarily popular in craft breweries, are made with the addition of fruit. Their production depends on the seasonality of fruit harvest and is influenced by the risk of microbial contamination due to microorganisms present on the fruit skin [51]. For centuries, fruits were used as beer ingredients, especially in Belgian lambic styles [52]. In Belgium, it is a tradition to add the whole fruit into the beer (lambic fermentation occurs in casks) for the production of cherry lambic (‘Kriek’) or raspberry lambic (‘Framboise’) [53]. In cherry lambic, 150–400 g/L intact sour cherries are added into wooden casks filled with a blend of old and young lambic beer, where the sugar from the fruits passes a secondary fermentation. Fruit addition during the fermentation process might enhance the content of bioactive compounds of beer, in particular polyphenols and carotenoids [54]. The fermentation process of lambics can be divided into four distinct stages: the *Enterobacteriaceae* phase, the primary fermentation phase, the acidification phase, and the maturation phase. Microorganisms are mainly responsible for the flavor and aroma within each stage [55]. *Hanseniaspora uvarum*, a wild yeast also known as *Kloeckera apiculata*, dominates the first stage of fermentation, while the second stage of the fermentation process is dominated by *Saccharomyces* spp. and in the third stage lactic acid bacteria (LAB). A wild yeast often associated with the spoilage of red wines and ciders, *Brettanomyces*, has positive effects upon flavor characteristics and dominates the final fermentation stage in lambic beer. The fermentation process starts after the wort has been cooled. *H. uvarum* rapidly reaches its maximum content of 10^5^ cells/mL within the first week of fermentation, but it is quickly overcome by *Saccharomyces. H. uvarum* can ferment glucose but not maltose or more complex carbohydrates. Consequently, pH drops from 5.1 to 4.6 due to the fast growth of *Enterobacteriaceae* and *H. uvarum* and the production of acetic and lactic acids. The following stages of fermentation overlap one another [56]. The third phase of traditional lambic beer production processes is characterized by the growth of lactic acid bacteria that are responsible for the acidification process, which is essential as lambic beer is recognized for its acidity [57]. Many of the lactic acid bacteria present in lambic beer are from the *Pediococcus* genus. Some strains have beneficial effects, and others can produce a “ropy” surface, creating permanent cloudiness [56,57,58]. The final stage of the process is marked by an increase in the number of yeast cells, *Brettanomyces* playing an essential role in developing the aromatic and flavor profile of lambic beer [56] by fermenting higher malto-oligosaccharides not fermented by *Saccharomyces.*

Cider is a fermented beverage with an alcohol level typically in the range 4–10% ABV [59] that recognizes ‘territoriality’ as essential for its appreciation [24]. Alcohol-free cider containing less than 0.5% ABV and low-alcohol cider, with an alcohol content between 0.5 and 1.2% ABV, are also marketed [60]. Traditionally, cider is made from apple, but it can also be made from other types of fruit such as wax apple (or Java apple, *Syzygium samarangense*), a tropical fruit having many nutritional bioactive compounds and high economic value [61]. Over time, Europe became known as the heart of cider production. In some countries (England, Spain, France, Ireland, and Germany), cider production is particularly abundant and dates back for centuries [60]. Smaller productions are found in countries such as Switzerland, Poland, and Austria [24]. According to the European Cider and Fruit Wine Association, in 2020, Romania was the first small market cider producer with 148.8 hL, followed by Portugal and Bulgaria with about 143 hL [62]. Cider production has also become an important and promising segment of the fruit industry in China, because of the large production of apples in this country [63]. The processing of apple cider involves the following steps: apple conditioning (washing and sorting); apple crushing (small pieces of 4–5 mm thickness); pressing and separating the apple juice; clarification and depectinization; yeast inoculation and alcoholic fermentation; addition of nutrients for the lactic acid bacteria (LAB) and malolactic fermentation; stabilization and maturation (i.e., wood aging, optional) [60]. During the process of fermentation and aging, sugars, acids, and pectic and phenolic substances present in the apples [64] are transformed into alcohols (propan-1-ol, iso-pentan-1-ol), organic acids (malic, lactic), and esters (ethyl and butyl lactate, ethyl-2 and ethyl-3-methylbutyrate, ethyl decanoate) [65]. Commercially, a selected strain of *Saccharomyces* spp. (*Saccharomyces cerevisiae)* [65] is used for the production of cider, but also other yeast strains were studied, such as *Hanseniaspora osmophila* and *Torulaspora quercuum* in co- and sequential fermentation, where the production of organic acids varied depending on the yeast species employed and inoculation method. The simultaneous fermentation resulted in the highest ethanol content because of a more efficient sugar consumption [66]. *Saccharomyces cerevisiae × Saccharomyces eubayanus* hybrids [67] were able to ferment the juice completely to about 5% ABV.

According to the International Organisation of Vine and Wine, wine is obtained through the alcoholic fermentation of grape juice by yeast, followed by the aging process. The term can be commonly used for all fermented beverages obtained from sweet fruits and vegetables, such as fruit wines, and undistilled alcoholic beverages [68] that are nutritive, flavorful, mild stimulants, and have an alcohol content ranging between 5–13% ABV [69]. These types of wines are made from blackberry (representing a traditional Croatian alcoholic beverage, a source of minerals and polyphenolics) [70], mulberry [71], pineapple and passion fruit [72], mango [73], and mixtures of banana, pawpaw, and watermelon [74]. The techniques used to obtain fruit wines are similar to those for the production of conventional wines. Some differences in the content of sugar and acids can be adjusted by diluting the juice or adding sugar, as well as by testing different yeast strains to select the most effective in different environments [68,72,75].

Kombucha is a refreshing low alcoholic beverage obtained from the fermentation of sweetened black or green tea, inoculated with acetic acid bacteria and osmophilic yeast (“tea fungus”). Given the ascending trends in kombucha consumption, its production was scaled up in recent years [76]. Its fermentation lasts between 7–21 days, and it is recognized for its beneficial nutritional and health effects [77]. Alternative raw materials for kombucha production are spices, fruits such as snake fruit [78] and papaya [79], fruit juices [80], leaves [81,82], coffee [83,84,85], vegetables [86], milk [87], and other food industry by-products and wastes [88].

Other fermented fruit beverages have a more limited diffusion, rendering them “minor” compared to globally known beverages such as vinegar, wine, beer, etc. However, based on raw materials typical of the area of origin, these minor beverages are essential expressions of local traditions. *Hardaliye* is an indigenous beverage of the Thrace region of Turkey and is produced from red grape juice fermented with crushed black mustard seeds [89]. *Ombike* is the generic name for a fermented beverage made from wild fruits, including bird plum, buffalo thorn, and makalani palm, prepared in Namibia, where *omalunga*, a palm wine [90], is also produced. *Tepache* is a popular beverage in Mexico, prepared by fermenting pineapple peel and sometimes adding other fruits such as orange, apple, and guava [91]. *Urwagwa* is an alcoholic beverage common in Rwanda, produced from the fermentation of bananas. In Botswana, *setopoti* and *khadi* are traditional alcoholic beverages made from the fermentation of watermelons and *Grewia flava* fruits, respectively [92].

Ultimately, fruit juice fortification with probiotics and/or prebiotics is a challenge and a frontier goal, as juices could combine their specific nutritional effects in addition to providing specific health benefits through probiotic bacteria or prebiotic ingredients [93].

## 2. Composition and Functional Potential of Fruit-Based Fermented Beverages

Alongside their nutritional properties, fruit-based fermented beverages may show a functional potential related to the content of bioactive compounds, in particular phenolics. The phenolic compounds of fruits can fortify or enhance food or beverage functionality [94], impacting their sensorial, nutritional, and antimicrobial proprieties [20]. Beverages such as fruit juice, beer, and wine are rich in hydroxycinnamic acids [95]. Table 1 shows the content of phenolic acids and flavonoids in some fruit-based fermented beverages. Quercetin, from the flavonol class, is found in fruits such as blueberries, strawberries, apple, goji, apricot, and grape and is recognized to have antioxidant, antidiabetic, and anti-inflammatory properties [96]. The flavonols and their derivatives significantly influence taste characteristics, particularly bitterness and astringency [97].

While vinegar has a complex composition of carbohydrates, organic acids, small traces of ethanol, acetoin, 2,3-butanediol, amino acids, and peptides, the most abundant organic acids are acetic, lactic, pyruvic, malic, and citric acid [98]. The acetification process has an impact on aroma volatiles, mainly due to oxidative reactions. Furthermore, monoterpenic alcohols are found in orange vinegar, esters in banana vinegar, C13-norisoprenoids in cherry vinegar, and lactones in mango vinegar, indicating that fruit vinegars have different aroma profiles depending on the type of fruit used for the production [44]. Moreover, hydroxymethylfurfural, synthesized by thermal decomposition of reducing sugars [99], has been detected in low amounts in wine vinegar and in concentrations up to 35 mg/L in cherry vinegar and some apple vinegars [100]. The consumption of vinegar has many health benefits, such as an attenuation of cardiac injury via anti-inflammatory and anti-adiposity actions [101], a reduction of body weight, body fat mass, and plasma triglyceride (TG) levels in obese patients [102], and anti-carcinogenic and antihypertensive effects [46]. Vinegar has been associated with diminished post-prandial glucose response following a high glycemic load meal [103] and presents high antioxidant activity, antimicrobial and antidiabetic effects, and therapeutic properties [33].

Regarding beer, many studies prove that the addition of fruit juice or fruit increases the content of phenolic acids, flavonoids, and resveratrol and, consequently, improves the antioxidant activity [54]. Still, a study on goji berries enriched amber ale beer showed an increase in bioactive compounds but did not improve the flavan-3-ol oligomers, which caused colloidal instability [50]. Cornelian cherry beer contains iridoids, which have cardiovascular, hypoglycemic, antihepatotoxic, choleretic, and antispasmodic activities [104]. Similarly, Cornelian cherry beer has high antioxidant properties. The best results, in terms of the degree of fermentation, were obtained when Cornelian cherry juice was added during the primary fermentation of the beer [105].

In fruit-based fermented beverages, the fermentation process enhances the quantity of B-group vitamins [106]. For instance, *Lactobacillus reuteri*, a Gram-positive bacterium, can thrive on several substrates and produce B12 vitamin and folate [107]. Santos et al. [108] tested the capability of this bacterium to ferment melon juice and obtained a 5-to 10-fold increase in folate amount. As for vitamin B12, it significantly increased from 0.28 to 11.47 mg/100 mL during fermentation of coconut water [109]. However, a decrease of vitamin C throughout the fermentation was observed in a fermented strawberry beverage (from 75.5 mg/100 g fruit to 34 mg/100 mL in strawberry wine) [110], as well as in fermented wax apple juice (from 4.97 mg/100 g to 3.81 mg/100 mL) [61] and fermented pineapple juice (4.52 mg/100 g to 2.95 mg/100 mL) [111].

## 3. Probiotic and Prebiotic Fruit-Based Fermented Beverages

According to the WHO, probiotics are defined as “live microorganisms able to give, when administered in adequate amounts, many health benefits to the host”. It is also considered that they have a beneficial effect upon health with a minimum of 10^9^ cells per daily dose [119]. Probiotic organisms must have the ability to resist gastric juices, exposure to bile, and proliferate and colonize the digestive tract. The most commonly used probiotic bacteria belong to the heterogeneous group of LAB (*Lactobacillus*, *Enterococcus*) and to the genus *Bifidobacterium* [120], while among yeasts, *Saccharomyces cerevisiae* var. *boulardii* is used [121]. Probiotics can increase the activity of enzymes, improve the intestinal barrier function, generate substances with antibacterial or bactericide effects, can modify the pH, modulate host immune function, intestinal carcinogenesis, cholesterol uptake, and competitive deprivation of pathogenic bacteria [122], alleviate lactose intolerance symptoms, atopic dermatitis symptoms in children, prevent the risk of allergy in infancy, and help with inflammable bowel disease (IBD) and irritable bowel syndrome (IBS) symptoms [123]. As a side effect, the long-term use of probiotics under antibiotic selection pressure could cause antibiotic resistance, and the resistance gene could be transferred to other bacteria [119].

Products that are traditionally considered the best carriers for probiotics are fermented milk products, but nowadays, up to 70% of the world population is affected by lactose intolerance, so a trend to obtain probiotic beverages from cereals, soybeans, and fruits and vegetables is observed. Fruit juices are generally preferred due to their natural content of essential nutrients [121]. The development of nondairy-based probiotic beverages has gained consumer attention due to the need for reducing cholesterol intake as well as frequent allergies/intolerances to dairy products [124,125]. Conversely, nondairy-based probiotic beverages improve the growth of beneficial bacteria, possess antimicrobial effects towards many foodborne pathogens [93,126], and have a pleasant aroma and taste [125]. The most commonly employed probiotics include different strains from *Lactobacillus* such as *L. acidophilus*, *L. helveticus*, *L. casei*, and *L. paracasei*, *Bifidobacterium* such as *B. bifidum*, *B. longum*, and *B. adolescentis*, and other species such as *Escherichia coli* Nissle, *Streptococcus thermophilus*, *Weissella* spp., *Propionibacterium* spp., *Pediococcus* spp., *Enterococcus faecium*, *Leuconostoc* spp., and *Saccharomyces cerevisiae* var. *boulardii* [93]. Commercially, the most preferred species for lactic acid fermented fruits are those belonging to the *genus Lactobacillus* and the genus *Bifidobacterium* [127].

Prebiotics are non-digestible food ingredients that can impart a beneficial effect to supplemented or indigenous colonic microbiota. According to the International Scientific Association for Probiotics and Prebiotics (ISAPP), a prebiotic ingredient has to meet three criteria: resistance to degradation by stomach acid, enzymes, or hydrolysis; fermentation by intestinal microbes; selective stimulation of the growth and/or activity of beneficial microorganisms in the gut [128]. Initially, prebiotics were seen as ingredients that could not be digested and positively affected the host by selectively stimulating the growth and/or activity of one or a limited number of bacterial species in the colon [129]. Prebiotics include dietary fiber, oligosaccharides such as inulin, and fructo- (FOS) and galacto-oligosaccharides (GOS). According to Global Market Insights, INC (Delaware, USA), the global prebiotic market is increasing and is expected to surpass USD 8.5 billion by 2024. From the technological point of view, the addition of prebiotics to foods and beverages improves sensory characteristics, such as taste and texture, and enhances the stability of foams, emulsions, and mouthfeel in a vast range of food applications, such as dairy and bakery products [130]. FOS have gained special attention due to their health benefits and sweet taste, very similar to that of sucrose. FOS stimulate the growth of *Bifidobacterium* spp. in the digestive tract, decrease serum lipids and total cholesterol, relieve constipations, and improve human health [131]. At low pH and high temperature, the most stable prebiotics are GOS, while inulin and FOS are less stable [132]. Consumption of prebiotics leads to several benefits, which include increased calcium absorption and enhanced bone mineralization during pubertal growth [129], alleviating the symptoms of IBS [132], protecting against Crohn’s disease, ulcerative colitis, and inducing weight loss [119].

Several studies have aimed at formulating fruit-based fermented beverages with prebiotic and probiotic activity (Table 2). It has to be highlighted, however, that the functional beverages have to be consumed in large amounts to benefit human health, as the bioactive compounds are found in small quantities, and the daily recommended dose is much higher (Table 3). For example, fermented pomegranate juice should be consumed 2.5 L daily to gain all the benefits of this product [133,134], while sea buckthorn wine should be consumed up to 20 L daily [134,135,136]. Therefore, there is a need for new strategies to improve the functionality of fruit-based fermented beverages.

## 4. Methods Used to Increase the Extraction Yield of Bioactive Compounds

### 4.1. Maceration and Decoction

Maceration and decoction are traditional extraction methods that require very simple equipment. Maceration is usually performed at room temperature [151,152], and it is the most employed technique for extracting compounds of interest from plant raw materials in the alcoholic beverage industry [153]. In small scale extraction, the steps used for maceration are grinding the plant material into small particles to increase the surface area, adding the solvent, and finally pressing out or filtrating. Shaking facilitates extraction by increasing diffusion and removing concentrated solution from the sample surface, for bringing new solvent and higher extraction yield [152]. Water, alcohol, or other solvents can be used, depending on the purpose. Wine, for example, is used in the preparation of vermouths, while vinegar might be enriched in bioactive compounds during or after acetic fermentation, with the maceration of herbal extracts or fruits [151]. Susana Gonzalez-Manzano et al. [154] showed that the extraction of flavan-3-ols during maceration reached the highest level at 12.5% ABV, and gallocatechin was primarily extracted from the skin and catechin from the seeds of the grapes.

Decoction consists of boiling a plant matrix in a specific volume of water for a defined time, then cooling and straining or filtering it. Lou et al. [155] observed that the total concentration of phenolics from *Crataegus pinnatifida*, an organic Chinese hawthorn berry, was significantly higher in decoction (4044–4148 μg/g) than maceration (3398–3792 μg/g). Decoction can provide better accessibility for extracting phenolics by disrupting the plant’s cell wall, releasing phenolic compounds that were conjugated or insoluble bounded.

### 4.2. Supercritical Fluid Extraction (SFE)

The process of extracting or separating one component from the matrix using supercritical fluids as solvents is one of the latest extraction technologies adopted in the industry, which can be used as a preparation step for further analyses or on a larger scale to eliminate unwanted material from a product (e.g., decaffeination) or collect a desired product (e.g., essential oils) [2]. Supercritical fluids (SCF) are produced by heating a gas above its critical temperature or compressing a liquid above its critical pressure and have solvent powers similar to a light hydrocarbon for most solutes. The most widely used gas in food industries is carbon dioxide. The wide range of physical conditions that can be applied make possible the preparation of very ‘light’ extracts, similar to essential oils, or more complex extracts, having a chemical composition comparable with that of the starting plant material, since the extraction process causes no thermal or chemical modifications [153].

Supercritical CO_2_ can be used for extracting many types of compounds, such as lycopene from watermelon [3], anthocyanins and phenolics from *Syzygium cumini* fruit pulp and jamun (Indian blackberry) [156], and imperatorin and meranzin from Citrus maxima peel [157]. Temperature and pressure both affect the extraction yield and have to be carefully tested and adjusted [3,156]. Supercritical CO_2_ can be used for removing ethanol from aqueous solutions and therefore has been proposed for developing a low-alcohol beverage from wine, maintaining the aroma and the antioxidant activity similar to that of the original wine [158]. The same technique has been also used for the stabilization of FOS-rich beverages, maintaining the prebiotic functionality [159].

### 4.3. Microwave-Assisted Extraction (MAE)

MAE is a combined extraction technique that uses traditional solvent extraction and the energy of microwaves. Microwave energy is used to heat solvents in contact with solid or liquid samples (or other fresh tissues), thereby partitioning the compounds of interest into the solvent. Microwaves heat the solution directly, and as a result, the temperature gradients are minimal, and the heating rate using microwave radiation is faster [4]. In the case of microwave extraction, mass ingredient and heat actions are targeted toward the exterior of the cells, enhancing the yields of extraction for high-value compounds while reducing the time needed to extract compounds. By comparison, in a conventional process, the heat transfer occurs from the heating medium to the inside of the cells [5].

MAE can be applied to extract several types of substances. For example, anthocyanins and polyphenols have been extracted from raspberry [160], catechin from *Arbutus unedo* L. fruits [6], pectin from dragon fruit peel [8], and polysaccharides from *Camptotheca acuminate* fruits, known as wild banana [10]. A comparison of MAE with maceration and ultrasound showed that MAE and maceration were the most effective methods, able to extract 1.7 mg/g and 1.4 mg/g catechin from *Arbutus unedo*, respectively. MAE reached the maximum extraction at 137 °C in 42 min.

Furthermore, the microwave pretreatment positively affects the total anthocyanin content of hawthorn beverages (from 1.314 mg/100 mL to 2.106 mg/100 mL) [161]. Positive effects of microwave-assisted extraction (1200 W for 10 min, 40 °C) were encountered when applied to the anthocyanin content of Merlot grape wines [13]. Microwaving a peach functional beverage for 1.5 min at 850 W:50 Hz of power determined the retention and stability of bioactive compounds for up to 30 days at 4 °C. It was observed that the pH and the cloud values of the processed juice reduced with storage time, the total soluble solids remaining almost consistent while the TPC decreased rapidly during the storage period [162].

### 4.4. Ultrasound Assisted Extraction (UAE)

As for ultrasound, it can enhance the extraction of nutraceuticals such as phenolics, anthocyanins, and aromatic compounds. The advantages of UAE include reduced extraction time, reduced thermal gradient, small equipment size, rapid control response, increased yield, and selective extraction [152,163].

Prakash Man et al. [9] studied the effects of temperature, power of the ultrasound, time, and solid–liquid ratio in the extraction of bioactive compounds from *Nephelium lappaceum* L. fruit peel. Increasing the temperature from 30 to 50 °C made the yield of anthocyanins and polyphenols increase due to the greater cavitation, as well as increased solubility and reduced viscosity, which facilitates the solvent to penetrate deeper into the matrix. The extraction yield increased when the duration began to increase from 10 to 20 min and then decreased when the duration was extended. The optimal parameters for the extraction were 20 W for 20 min, a solid–liquid ratio of 1:18.6 g/mL, 50 °C, when were obtained 10.17 mg/100 g of the total anthocyanin content, 100.93 mg rutin equivalent (RE)/100 g total flavonoid content and 546.98 mg GAE/100 g TPC, respectively. The same fruit peel was also used to extract polysaccharides using ultrasound-assisted extraction. The highest extraction yield for polysaccharides was 8.31%, obtained at an extraction duration of 41 min, 110 W, 53 °C, and a solid–liquid ratio of 1:32 g/mL [7].

Ultrasound, alone and/or in combination with other techniques, was reported to affect *E. coli* and *Listeria monocytogenes* in apple cider. Moreover, the quality of fruit juice, such as orange, guava, and strawberry, has been affected on a smaller scale [164].

The effect of ultrasonication, pulsed light, and their combined usage on the phenolic profile and the antioxidant activities of lactic-acid-fermented mulberry juice has been assessed [112]. The mulberry juice was ultrasonicated for 15 min, 28 kHz, 60 W, at 5 °C. As for the pulse light treatment, the sample was subjected to light pulses at a pulse width of 360 μs, a frequency of 3 Hz, and delivering radiant energy of 1.213 Jcm^−2^ pulse^−1^. An exposure time of 4 s to obtain a sub-lethal energy dose of 7.26 Jcm^−2^ was employed. The TPC and total anthocyanin concentration significantly increased in the fermented mulberry juice treated with pulsed light and ultrasound.

### 4.5. Enzyme Assisted Extraction (EAE)

Enzymatic extraction of bioactive compounds from plants is an alternative to conventional solvent-based extraction methods. Enzymes are ideal for assisting in extracting, synthesizing, and modifying complex bioactive compounds of natural origin. This method is based on the inherent ability of enzymes to catalyze reactions with exquisite specificity, region selectivity, and an ability to function under mild processing conditions in aqueous solutions. In general, enzymes have been used to treat the plant material before conventional extraction methods. Various enzymes, such as cellulases, pectinases, and hemicellulases, are used to enhance the extraction yield of bioactive compounds from plants by altering the integrity of the cell wall and increasing permeability. In food processing, pectic enzymes are employed industrially for the extraction, clarification, and concentration of fruit juices and to extract several compounds from plant materials [15]. The addition of pectinase as a pre-treatment in red dragon fruit juice, followed by fermentation with *Torulaspora delbrueckii*, increased the beverage yield by 16% and enhanced the levels of esters and terpenes, improving aroma profile. Other effects included higher acetic acid production and impairment on the yeast’s ability to metabolize the nitrogen compounds, with a loss of color intensity and a decrease of pigments [165].

The effects of the EAE on the stability of bilberry (*Vaccinium myrtillus* L.) anthocyanins in chilled storage at 10 °C for 9 days and extractability yield were tested by Dinkova et al. [166]. The results showed an increase of 43–60% in polyphenol content and 23–37% in anthocyanins, while the antioxidant capacity increased by 35% through enzymatic maceration. A decrease in counts of mesophilic and psychrotrophic microorganisms was also observed, as well as that of molds and yeasts at the end of storage, suggesting an inhibition of the microbial growth. The same method, by adding pectinase, was applied to extract anthocyanins from blackberry. An increase in extraction yield was observed with the increase of enzyme loading. Yield also increased by increasing the temperature up to 50 °C and then decreased due to enzyme inactivation [16]. EAE has also been used to extract polysaccharides from *Cornus officinalis* fruits, at an enzyme concentration of 2.15%, pH 4.2, 55 °C, for 97 min [17]. Moreover, pectinase has been used in the preparation of pear juice made from William Bartlett cultivar [18]. The juice yield ranged between 67.62–90.57% and the clarity from 38–91.1%. The clarity increased significantly with the enzyme concentration and the best results were obtained at 1.9% enzyme concentration, 30 °C, and 2 h incubation. Some disadvantages of EAE, however, are the high price of enzymes, and difficulties in the industrial scale-up, due to different environmental conditions for every enzyme [15].

By using commercial enzymes, Kim and Park [167] enhanced the volatile aromatic compound from Korean black raspberry wines. The wine, treated with a mix of pectinase, cellulose, hemicelluloses, and β-glucosidase, had the highest concentration of aromatic compounds: terpenes 103.1 mg/L, esters 461 mg/L, and the score of fruity and floral aroma in the sensory evaluation.

### 4.6. Pulsed Electric Field (PEF)

PEF is a novel non-thermal technology that causes the degradation of nutritional and sensory characteristics to a lesser extent than traditional thermal processing. It uses an electrical field in the form of short- or high-voltage pulses applied to a food item placed between two electrodes for a short time, usually in the microsecond scale [168]. The PEF can be used to control the sugar/ethanol conversion rate during fermentation, solving issues caused by some techniques used to reduce the alcohol content in beverages. This is due to the high sugar content in fruits, intensified by global warming, the fermentation of early-harvested fruits, and many such similar matters that have a low impact on the ethanol concentration of the beverage. They may influence the end result by modifying the aroma and mouthfeel of the product [169], stimulating alcoholic fermentation and S. cerevisiae growth while applying direct current (10 mA), alternative current (100 Ma), and electrical potential (0.75 V) to the must [170].

Siddeeg et al. [171] observed the impact of PEF application on the free amino acids, physical and chemical characteristics, and bioactive compounds of alcoholic beverages obtained from date palm fruits. The permeabilization of plant cells can be useful for more effective extraction of bioactives from fruit. The TPC increased from 3.50 mg CE/100 g to 91.90 mg CE/100 g in the highest PEF, while the total carotenoid content increased from 3.10 to 5.15 µg/mL. A higher yield of anthocyanins was revealed in PEF treatment (0.7 kV/cm, for 200 ms and 31 Wh/kg) of Cabernet Sauvignon red grapes [172].

Moderate PEF coupled with *Hanseniaspora* sp. yeast treatment during cider production significantly increased biomass growth and decreased ethanol yield. PEF can also be used as an alternative to thermal pasteurization of beverages, as it solely modifies a small part of the sensory characteristics. Temperature increases with PEF are low and typically not more than 40 °C, but cooling systems can be attached to the standard equipment if needed. Furthermore, PEF destroys microorganisms by modifying the electric potentials between each side of the membrane [173].

### 4.7. High Pressure Homogenization (HPH)

HPH is a mechanical process that works to reduce particle size or to lyse cells. Rheological changes have been observed in mango juice after the use of HPH, and in kiwifruit purée HPH improved the appearance of cloudy apple juice to increase consumer acceptability. This technique improved the uniformity and cloud stability due to the reduced size of particles, increased viscosity, and yield stress. The added purée reduced enzymatic browning due to the ascorbic acid creating a saturated and bright yellow color and an improved sensory quality [174]. Morata et al. [175] proved that HPH treatment above 400 MPa applied for 10 min completely destroyed the yeasts.

Similarly, heating and high HPH treatment on litchi juice showed a good ability of HPH to preserve color, flavor acceptance, and antioxidant activity. Moreover, HPH and the heating process offered the opportunity to improve the viability of probiotic cells and extend product quality attributes after 4-week storage at 4 °C [176].

Positive results were also reported on a sulfur dioxide-free wine after using HPH treatments at 500 MPa [177]. The wine presented more significantly the scent of cooked fruit and a spicy aroma, while the untreated wine presented a more pronounced metallic and leathery aroma and a less perceived fruity and floral aroma. After 12 months of storage, the pressurized wine presented notes of an aged wine. The results demonstrated that HPH can influence long-term red wine physic-chemical and sensory characteristics.

However, despite the numerous advantages of recent technologies in obtaining added-value fermented fruit beverages, there are also some disadvantages that beverage industry stakeholders have to consider, which are synthesized in Table 4.

## 5. Fermentation Types to Enhance the Functionality Potential

### 5.1. Alcoholic Fermentation

Among fermented beverages, wine results from the complex interactions between yeasts, bacteria, and other fungi that start in vineyards and continue with the fermentation process in the winery. Different yeast species are predominant on the surface of grape skins and in the winery environment, *Saccharomyces cerevisiae* being the main species responsible for this process. In recent years, non-Saccharomyces species with fermentative abilities were studied, emphasizing their critical role in wine fermentation under specific controlled conditions [201].

Benito et al. [202] studied the possibility of producing red wine using *Schizosaccharomyces*, which consumed less primary amino nitrogen and had less urea and more pyruvic acid than the *Saccharomyces* strains.

Interestingly, melatonin, a hormone that modulates physiological processes in humans such as circadian rhythms and the reproductive function, has been recently detected in wine, and its presence has been linked to yeast activity in the fermentation process [203], which was also confirmed by Rodriguez-Naranjo et al. [204], who showed that melatonin is absent in grapes and musts and is formed during alcoholic fermentation. As melatonin is also found in the orange fruit, Fernández-Pachón et al. [205] measured the melatonin content during the alcoholic fermentation of orange juice and revealed that melatonin content significantly increased throughout fermentation from day 0, 3.15 ng/mL, until day 15 when it reached 21.8 ng/mL. Compared to other melatonin contents in fruits, such as banana 0.47 ng/g, apple 0.05–0.16 ng/g, pineapple 0.30 ng/g, and Primoris strawberry 11.26 ng/g [206], the fermented orange juice can be a promising source of this bioactive compound. It can also provide a rich source of carotenoids and has health effects similar to those of the orange juice, with a higher content of total carotenoid and provitamin A (6.65 mg/L and 90.57 retinol activity equivalents per liter of juice (RAEs/L) than the non-fermented orange juice (5.37 mg/L and 75.32 RAEs/L) [207]. Moreover, the potential bioavailability of flavonoids increases during fermentation due to the high content of hesperetin-7-O-glucoside, which is twofold higher at the end of the fermentation process; the same ascendant trend was observed for the antioxidant activity [208].

### 5.2. Acetic Fermentation

Acetic acid bacteria (AAB) are Gram-negative, rod-shaped, obligate aerobes commonly known as vinegar-producing microorganisms. The AAB, in general, can oxidize ethanol to acetic acid and glucose to gluconic acid [209] and are currently found based on phylogeny, physiology, and ecology in the *Acetobacteraceae* family, class *Alphaproteobacteria* [210]. AAB are often found on flowers, plants, and fruits, as these aerobic environments are rich in carbohydrates, sugar alcohols, and/or ethanol, allowing a specific respiratory chain to rapidly and incompletely oxidize these substrates into organic acids for energy production. Acidification of the environment prevents the growth of competitive bacteria. These physiological characteristics explain the occurrence of AAB and underline their role in producing diverse fermented foods and beverages such as lambic beer, water kefir, kombucha, and cocoa [57]. The species most frequently reported in vinegar production comprise Acetobacter species such as *Acetobacter cerevisiae*, *A. malorum*, and *A. pasteurianus*, *Gluconacetobacter* species such as *G. entanii*, *G. liquefaciens*, and *Gluconobacter oxydans*, and *Komagataeibacter* species such as *K. hansenii*, *K. intermedius*, *K. medellinensis*, and *K. oboediens* [211].

Acetic fermentation is also an excellent method for transforming by-products into value-added products. Giuffrè et al. [212] used acetic fermentation to valorize bergamot (*Citrus bergamia*) peel and juice. Limonene, a compound exhibiting antioxidant activity, was found in high contents (0.4–2.04 mg/L) in the vinegar obtained. The TPC varied from 241 to 1953 mg/L and chlorogenic acid between 2.95–58.51 mg/L.

### 5.3. Lactic Fermentation

*Leuconostoc* spp., *Lactococcus* spp., *Lactobacillus* spp., and *Streptococcus thermophilus* are examples of LAB that can convert sugars into lactic acid. Lactic acid inhibits the growth of subsequent and potentially harmful bacteria of other species and creates favorable conditions for yeast activity, a property utilized in the production of wine and beer [213]. In winemaking, LAB are present throughout all stages, and they can either enhance or diminish the quality of wine through malolactic fermentation and affect the organoleptic properties of the final product. The concentration of LAB during malolactic fermentation reaches approximately 10^7^ CFU/mL, which indicates their importance in winemaking. The density of LAB in the initial phases of winemaking ranges from about 10^3^ to 10^4^ CFU/m. The most abundant are *Lactobacillus hilgardii*, *L. plantarum*, *L. casei*, *Leuconostoc mesenteroides*, and *Pediococcus damnosus*, while less common species include *Oenococcus oeni* and *Lactobacillus brevis* [214].

Lactic fermentation can be used in the beverage industry to enhance antioxidant and antimicrobial properties or produce probiotic beverages. Muhialdin et al. [215] tested the effect of dragon fruit juice fermentation with *L. plantarum* on consumer acceptability and biological activity of the final product. The results show that fermentation at 37 °C for 48 h enhanced the antibacterial activity of fermented dragon fruit juice threefold. As per acceptability, consumers preferred the mixed dragon fruit fermented juice with fresh juice in the 1:9 ratio, as the fermented juice was too sour, with low pH (3.49). Additionally, this beverage had an extended shelf life to 3 months with no detected odors, color, and taste modifications compared to the fresh dragon fruit juice, which developed an unpleasant aroma and taste only after 1 month of storage at 8 °C.

LAB can be used to prolong shelf life up to six months for fresh cantaloupe juice [216]. The juice was fermented with *L. plantarum* for 48 h. After fermentation, the antimicrobial activity increased due to several bioactive metabolites and demonstrated complete inhibition towards *Aspergillus flavus*. The ratio of 20:80 fermented cantaloupe juice to fresh juice showed stable shelf life and high consumer acceptability.

A significant increase in the concentration of ellagic acid, known as a potent antioxidant and anti-tumor compound [217], was noticed during the fermentation of pomegranate juice by *L. plantarum*, which can degrade tannins by a tannin acylhydrolase named tannase, inhibiting the polymerization of the aromatic compounds and juice browning. Tannase can also modify the phenolic composition of juices and improve their antiproliferative and antioxidant activities [20]. Papaya juices fermented by *L. acidophilus* and *L. plantarum* were compared in organic acids, volatile compounds, and antioxidant capacities [218]. The DDPH and ABTS radical scavenging activities of *L. plantarum* fermented juice were significantly higher than *L. acidophilus* fermented juice.

### 5.4. Symbiotic Fermentation

Symbiosis, or the coexistence of various microorganisms, such as LAB and AAB or yeasts and molds, contributes to traditional fermentation (brewing). Auxotrophy and optimum oxido-reduction potential of microorganisms are essential factors for their symbiotic interaction. As for LAB, they usually require different types of nutrients, such as amino acids and vitamins. In traditional fermentation environments, some nutrients are provided to LAB using yeasts and kōji molds. LAB and yeasts usually favor anaerobic environments, while kōji molds and AAB absolutely require oxygen for growth. Therefore, in traditional fermentation environments, kōji molds and AAB are usually found on surface areas, and LAB and yeasts are typically localized in internal areas [219].

Symbiosis in the forms of mutualism or commensalism is widespread in fermented foods, for example, in yoghurt, milk kefir, or sourdough [220]. A type of symbiotic fermentation is one that uses kefir grains. Kefir grains are white or yellow irregular granules of protein and a polysaccharide matrix named kefiran. The starter culture consists of a symbiotic consortium of several yeasts and bacteria. LAB are represented by the genera of *Leuconostoc*, *Lactobacillus*, and *Streptococcus*; the yeasts include *Saccharomyches*, *Zygosaccharomyces* spp., *Candida*, *Pichia*, and *Dekkera* [20]. To date, a great variety of fruits such as pomegranate [221], apple [222], and orange [223] have been used to produce functional beverages using kefir grains.

Similarly, a symbiotic culture of bacteria and yeasts (SCOBY) is used for the production of kombucha. SCOBY mainly contains acetic acid bacteria (AAB), lactic acid bacteria (LAB), and yeasts. *Komagataeibacter* (*K. xylinus*, *K. kombuchae*), *Acetobacter* (*A. aceti*, *A. pasteurianus*, *A. nitrogenifigens*), and *Gluconacetobacter* (*G. sacchari*) are major AAB strains, while strains of *Saccharomyces* (*S. cerevisiae*) and non-*Saccharomyces* (*Candida* spp., *Schizosaccharomyces* spp., *Dekkera* spp., and *Brettanomyces* spp.) are present in lower amounts. Recent research successfully tested the isolates of *Hanseniaspora valbyensis*, *Hanseniaspora vineae*, *Torulaspora delbrueckii*, *Zygosaccharomyces bailii*, and *Zygosaccharomyces kombuchaensis* [150] and *Lachancea fermentati* strains [224] from kombucha to produce alcohol-free beer and low alcohol beer, respectively.

Another example of a symbiotic product is a type of plum juice containing three different types of probiotics (*Lactobacillus kefiranofaciens*, *Candida kefyr*, and *Saccharomyces boulardii*) and oligosaccharides, which showed antibacterial activity against diarrhea-causing pathogens such as *Escherichia coli*, *Vibrio cholerae*, *Salmonella* Paratyphi A, *Shigella dysenteriae*, and *Staphylococcus aureus* [225]. In this range, another symbiotic beverage has been formulated using probiotics and prebiotics. As a raw material, coconut water was used, *Lactobacillus rhamnosus* as a probiotic, and inulin as a source of soluble fiber. The obtained beverage presented good sensory acceptance, an amount of 82 × 10^8^ CFU/mL of *L. rhamnosus*, 15 g of inulin, and a shelf life of 15 days in refrigeration conditions.

## 6. Future Trends and Conclusive Remarks

One of the emerging trends in the food and beverage industry is reusing by-products and waste, in the perspective of a circular economy. Fruit-based fermented beverages, a convenient and rich source of bioactive compounds, can work as carriers to deliver health-benefiting components derived from fruit by-products [226]. The management of fruit waste and/or by-products is essential because they represent a valuable resource of compounds that can be reused (phenolic compounds, carotenoids, tocopherols, and dietary fibers) [227]. Waste or by-products can also be used as raw material, decreasing the volume of food waste accumulated in landfills [228]. Studies have demonstrated an improvement in TPC and/or antioxidant activity by using by-products from pomegranate, orange, banana, grape, and tamarind in beverages. For instance, a beverage made from fresh orange juice with added banana peel extract (500 mg/100 mL) reported a 21 and 150% increase for DPPH and FRAP assays, respectively, compared to the non-fortified juice [226]. Moreover, pineapple residues (100 g/kg peel) can be used to produce fermented alcoholic beverages without quality loss or negative interference with the chemical quality of the fermented products [228].

In conclusion, fermentation has shown excellent potential for the production of fruit-based beverages directly from fruits or even from their waste and by-products. Optimized fermentation processes allow for enrichment in specific bioactive compounds (typically phenolic compounds and vitamin C), serving the interest of both industry and consumers. Furthermore, current emerging technologies, based on supercritical fluids, microwaves, ultrasound, enzymes, PEF, and HPH, represent another tool to increase the extraction yield of bioactive compounds.

The perception that beverages made with natural ingredients are healthier is increasing among European consumers. It is imperative to address fermented fruit-based beverages, considering consumer trends, preferences, and needs. Moreover, there is a great interest globally in consuming functional non-dairy fermented beverages. Recent studies have proved the functionality of fruit-based beverages, based on each nutritional or functional compound (namely phenolic compounds, vitamins, prebiotics, and probiotics). Therefore, these beverages represent a convenient, easily assimilable source that can be integrated within a daily diet.

## Figures and Tables

**Table 1:** Concentration of the main phenolic compounds found in different fruit-based fermented beverages.

Beverage	Phenolic Compounds (μg/mL)								References
	Phenolic Acids					Flavonoids			
	Gallic Acid	Caffeic Acid	Ferulic Acid	*p*-Coumaric Acid	Chlorogenic Acid	Catechin	Rutin	Quercetin	
Fermented mulberry juice	15	98.3	187.5	2.4	91.6	163.5	194.7	300.8	[112]
Wax apple cider	11.5	62.1	69.1	0.14	64.6	110.1	41.3	61.1	[61]
Cider with added apple pomace	3.9	3–3.7	2–4.2	1–1.5	8.3–22.5	24.4	1.8–3.3	2.1	[63,113,114]
Amber ale beer enriched with goji berries	0.01–5.9	1.3	4.6–13.3	1.19–3.7	0.01–7.8	0.8–6.9	23.1	320–1400	[50,115]
Blackberry wine	4.5–118	1.2–10.1	1.3	0.3–4	1.2–8.3	9–45	35.1	34.9	[70,116,117,118]

**Table 2:** Prebiotic and probiotic content in fruit-based fermented beverages.

Prebiotic Added	Recommended Dose for Fortifying	Beverage Type	Content of Prebiotic and Probiotic Compound	References
Inulin	3% *w*/*w* for infant formulas and 5% *w*/*w* for common beverages 5 g in Europe 9–10 g in Korea 1.25 g/portion in Southeast Asia	*L. rhamnosus* probiotic orange juice fortified with long chain inulin	*L. rhamnosus* 5.9 × 10^7^ CFU/mL; long chain inulin 4 g/100 mL	[137,138,139,140]
		Pineapple juice enriched with *L. casei* or *L. rhamnosus* and inulin	*L. casei* (10^8^ CFU/mL) or *L. rhamnosus* (10^8^ CFU/mL); inulin 2 g/100 mL	
		Fig juice fortified with inulin and *L. delbrueckii*	*L. delbrueckii* ˃10^6^ CFU/g; inulin 2 g/100 mL	
GOS	Max 5% *w*/*w* in beverage	PS-enriched milk-based fruit beverage with 5 g GOS/250 mL	GOS 2 g/100 mL	[141,142]
FOS	5 g in Europe 1.25 g/portion in Southeast Asia 3% *w*/*w* for infant formulas and 5% *w*/*w* for common beverages	FOS-fortified apple juice	FOS 15.5 g/100 mL	[137,143,144]
		FOS-enriched mixed fruit beverage	FOS 0.7 g/100 mL	
		Prebiotic orange juice	FOS 7 g/100 mL	

**Table 3:** Content of the main compounds of interest, daily recommended dose, and health effects of fermented beverages from different fruits.

Type of Beverage	Fermentation Type	Chemical Compound/Class of Compounds of Interest	Concentration of Compounds of Interest	Daily Recommended Dose of Compound of Interest	Impact on Health	References
Grape-based fermented beverage	Lactic	Polyphenols (grape must), γ-amino butyric acid *L. plantarum* DSM19463	γ-amino butyric acid 10 g/20 mL; Log 10.0 ufc/g *L. plantarum*	γ-amino butyric acid: 10 g	Anti-hypertensive activity; anti-inflammation and fibroblast cell proliferation, activities that promote the healing process of wounds	[145]
Fermented jabuticaba berry beverage (12% ABV)	Alcoholic	Phenolic compounds (rutin, quercetin, anthocyanins, coumarins, gallic acid)	Gallic acid 3 mg/mL; phenolics and coumarins 68.9 mg/mL	Quercetin: 13.4–22.8 mg—men; 11.1–17.7 mg—women	Antioxidant, vasorelaxation, and cardiovascular protection	[134,146]
Pomegranate fermented juice	Lactic	Phenolic compounds (ellagic acid) *L. plantarum*	Ellagic acid 6.4–7.1 mg/L; *L. plantarum*: 6 Log CFU/mL	Ellagic acid: 17.9 mg—men; 27.6 mg women (USA)	Antioxidant and anti-inflammatory proprieties, inhibits growth of tumor cells	[133,134]
Sea buckthorn wine	Alcoholic	Rutin, myricetin, quercetin, vitamin C	Ascorbic acid 176.8 mg/L; rutin 68.4 mg/L; myricetin 40.3 mg/L; quercetin 1.04 mg/L	Quercetin: 13.4–22.8 mg—men; 11.1–17.7 mg women Vitamin C: 90 mg—men, 75 mg—women	Protective effects against oxidative stress and hypercholesterolemia; anti-inflammatory, myocardial protecting, vasodilator and hepatoprotective activities	[134,135,136]
Symbiotic fermented Cornelian cherry beverage (0.2–0.7% ABV)	Alcoholic and lactic	Fibers, phenols, *L. paracasei* K5, lactic acid	Wheat bran-50% dietary fiber (used as a prebiotic and cell immobilization carrier for *Lactobacillus*) *L. paracasei* 9.74 Log CFU/mL, lactic acid 167.8 mg/100 mL	10^10^–3 × 10^12^ CFU *L. paracasei*	Antimicrobial activity against various pathogens; anti-inflammatory, antimalarial, antidiabetic activities	[147,148,149]
Symbiotic fruit based kombucha beverage	Acetic, lactic and alcoholic	Organic acids, vitamins, phenols, minerals, probiotics, carbohydrates, amino acids, bacteriocins	Acetic acid 0.016–39 g/L, gluconic acid 0.18 g/L, vitamin B1 0.74 mg/mL, vitamin B2 0.08 mg/mL, vitamin B6 0.52 mg/mL, vitamin B12 0.84 mg/mL, vitamin C 25,000 mg/mL Cu, Fe, Mn, Ni, Zn 0.1 to 0.4 μg/mL Total phenolic content 178–264 mg GAE/L Flavonoid content 2307.14 mg QE/L	Vitamin C: 90 mg—men, 75 mg—women	Antimicrobial, antioxidant, anti-inflammatory activities, anticarcinogenic potential	[77,78,136,150]

**Table 4:** Advantages and disadvantages of methods to improve the functional potential of fruit-based fermented beverages.

Methods	Type	Advantages	Disadvantages	References
Pulsed electric field (PEF)	Non-thermal	Reduction of the quantity of SO_2_ used Reduced degradation of nutritional and sensorial characteristics Controlled sugar/ethanol conversion in fermentation Improved extraction of compounds of interest Alternative to pasteurization Inactivation of microorganisms	Corrosion and migration of electrode materials Metallic taste Degradation of some compounds (cyanidin-3-O-glucoside and cyanidin-3-O-sophoroside)	[168,169,170,171,172,173,178,179]
Enzyme assisted extraction (EAE)	Non-thermal	Improved yeast growth by releasing nutrients Enhanced aroma of the beverage Increased yield of fermentation Improved clarification process and biological activity	Susceptible to substrate or product inhibition The product may cause an allergic reaction High cost for isolation and purification Difficult recovery for subsequent reuse Higher temperatures (˃40 °C) and pH over 7.4 lead to denaturation	[19,161,165,167,180,181,182,183,184]
Microwave assisted extraction (MAE)	Thermal	Increased total anthocyanin content Enzyme inactivation Inactivation of microorganisms Increased juice stability	Only certain microwave frequencies can be used because they can interfere with radio frequencies Non-uniform temperature distribution resulting in hot and cold spots Longer extraction step and poor extraction yield	[11,12,13,162,185,186,187,188]
Ultrasound assisted extraction (UAE)	Non-thermal	Increased content of bioactive compounds Prevention of browning Shortened aging process Inactivation of microorganisms	Degradation of ascorbic acid Decreased content of minerals Increased ultrasonic intensities may cause physical-chemical degradation	[112,164,189,190,191,192,193,194]
High pressure homogenization (HPH)	Non-thermal	Improved yield of extraction Increased shelf life Inactivation of microorganisms Changed rheological characteristics Improved overall aspect of beverages, especially color	Pressures exceeding 1200 MPa are needed to inactivate bacterial spores Most products must be stored and transported under refrigeration *Cladosporium* spores may survive in low-acid HPH products. High processing costs	[174,175,176,177,195,196,197,198,199,200]

## Data Availability

Not applicable.

## References

[1] Laureys D., De Vuyst L. (2014). Microbial species diversity, community dynamics, and metabolite kinetics of water kefir fermentation. Appl. Environ. Microbiol..

[2] Sapkale G.N., Patil S.M., Surwase U.S., Bhatbhage P.K. (2010). Supercritical Fluid Extraction: A Review. Int. J. Chem. Sci..

[3] Vaughn K., Clausen E., King J., Howard L. (2008). Extraction conditions affecting supercritical fluid extraction (SFE) of lycopene from watermelon. Bioresour. Technol..

[4] Llompart M., Garcia-Jares C., Celeiro M., Dagnac T. (2019). Extraction|Microwave-Assisted Extraction, Encyclopedia of Analytical Science.

[5] Gomez L., Tiwari B.K., Garcia-Vaquero M., Torres M.D., Kraan S., Dominguez H. (2020). Emerging extraction techniques: Microwave-assisted extraction. Sustainable Seaweed Technologies.

[6] Albuquerque B.R., Prieto M.A., Barreiro M.F., Rodrigues A., Curran T.P., Barros L., Ferreira I.C.F.R. (2017). Catechin-based extract obtained from *Arbutus unedo* L. fruits using maceration/microwave/ultrasound extraction techniques. Ind. Crop. Prod..

[7] Maran J.P., Priya N. (2014). Ultrasound-assisted extraction of polysaccharides from *Nephelium lappaceum* L. fruit peel. Int. J. Biol. Macromol..

[8] Maran J.P., Sivakumar V., Thirugnanasambandham K., Sridhar R. (2014). Microwave assisted extraction of pectin from waste *Citrullus lanatus* fruits rinds. Carbohydr. Polym..

[9] Maran J.P., Manikandan S., Nivetha V.C., Dinesh R. (2017). Ultrasound assisted extraction of bioactive compounds from *Nephelium lappaceum L.* fruit peel using central composite face centered response surface design. Arab. J. Chem..

[10] Hu W., Zhao Y.Z., Yang Y., Zhang H., Ding C., Hu C., Zhou L., Zhang Z., Yuan S., Chen Y. (2019). Microwave-assisted extraction, physicochemical characterization and bioactivity of polysaccharides from *Camptotheca acuminata* fruits. Int. J. Biol. Macromol..

[11] Perez-Grijalva B., Herrera-Sotero M., Mora-Escobedo R., Zebadua-Garcia J.C., Silva-Hernandez E., Oliart-Ros R., Perez-Cruz C., Guzman-Geronimo R. (2018). Effect of microwave and ultrasound on bioactive compounds and microbiological quality of blackberry juice. LWT.

[12] Siguemoto E.S., Gut J.A.W., Martinez A., Rodrigo D. (2018). Inactivation kinetics of *Escherichia coli* O157:H7 and *Listeria monocytogenes* in apple juice by microwave and conventional thermal processing. IFSET.

[13] Casassa L.F., Sari S.E., Bolcato E.A., Fanzone M.L. (2019). Microwave-Assisted Extraction Applied to Merlot Grapes with Contrasting Maturity Levels: Effects on Phenolic Chemistry and Wine Color. Fermentation.

[14] Chan C.-H., Yusoff R., Ngoh G.-C., Wai-Lee Kung F. (2011). Microwave-assisted extractions of active ingredients from plants. J. Chromatogr. A.

[15] Puri M., Sharma D., Barrow C.J. (2011). Enzyme-assisted extraction of bioactives from plants. Trends Biotechnol..

[16] Gong H., Qi L., Yang Z. (2014). Optimization of enzyme-assisted extraction of anthocyanins from blackberry (*Rubus fruticosus* L.) juice using response surface methodology. Afr. J. Pharm. Pharmacol..

[17] You Q., Yin X., Zhao Y. (2013). Enzyme assisted extraction of polysaccharides from the fruit of *Cornus officinalis*. Carbohydr. Polym..

[18] Gani G., Naik H.R., Jan N., Bashir O., Hussain S.Z., Rather A.H., Reshi M., Amin T. (2021). Physicochemical and antioxidant properties of pear juice prepared through pectinase enzyme-assisted extraction from William Bartlett variety. J. Food Meas. Charact..

[19] Claus H., Mojsov K. (2018). Enzymes for wine fermentation: Current and perspective applications. Fermentation.

[20] Ayed L., M’hir S., Hamdi M. (2020). Microbiological, Biochemical, and Functional Aspects of Fermented Vegetable and Fruit Beverages. J. Chem..

[21] Melini F., Melini V., Luziatelli F., Ficca A.G., Ruzzi M. (2019). Health-Promoting Components in Fermented Foods: An Up-to-Date Systematic Review. Nutrients.

[22] Feng Y., Zhang M., Mujumdar A.S., Gao Z. (2017). Recent research process of fermented plant extract: A review. Trends Food Sci. Technol..

[23] Fiorda F.A., de Melo Pereira G.V., Thomaz-Soccol V., Rakshit S.K., Pagnoncelli M.G.B., Vandenberghe L.P.S., Soccol C.R. (2017). Microbiological, biochemical, and functional aspects of sugary kefir fermentation—A review. Food Microbiol..

[24] Cousin F.J., Le Guellec R., Schlusselhuber M., Dalmasso M., Laplace J.-M., Cretenet M. (2017). Microorganisms in fermented apple beverages: Current knowledge and future directions. Microorganisms.

[25] Behera S.S., Panda S.K. (2020). Ethnic and industrial probiotic foods and beverages: Efficacy and acceptance. Cur. Opin. Food Sci..

[26] Anal A.K. (2019). Quality ingredients and safety concerns for traditional fermented foods and beverages from Asia: A review. Fermentation.

[27] Žuntar I., Petric Z., Bursać Kovačević D., Putnik P. (2020). Safety of probiotics: Functional fruit beverages and nutraceuticals. Foods.

[28] Rizo J., Guillén D., Farrés A., Díaz-Ruiz G., Sánchez S., Wacher C., Rodríguez-Sanoja R. (2020). Omics in traditional vegetable fermented foods and beverages. Crit. Rev. Food Sci. Nut..

[29] Chaiyasut C., Sivamaruthi B.S., Makhamrueang N., Peerajan S., Kesika P. (2018). A survey of consumer’ opinion about consumption and health benefits of fermented plant beverages in Thailand. Food Sci. Technol..

[30] Salanță L.C., Coldea T.E., Ignat M.V., Pop C.R., Tofană M., Mudura E., Borșa A., Pasqualone A., Anjos O., Zhao H. (2020). Functionality of special beer processes and potential health benefits. Processes.

[31] Jimborean M.A., Salanță L.C., Trusek A., Pop C.R., Tofană M., Mudura E., Coldea T.E., Farcaș A., Ilieș M., Pașca S. (2021). Drinking behavior, taste preferences and special beer perception among Romanian university students: A qualitative assessment research. Int. J. Environ. Res. Public Health.

[32] Mangindaan D., Khoiruddin K., Wenten I.G. (2018). Beverage dealcoholization processes: Past, present, and future. Trends Food Sci. Technol..

[33] Ho C.W., Lazim A.M., Fazry S., Zaki U.K.H.H., Lim S.J. (2017). Varieties, production, composition and health benefits of vinegars: A review. Food Chem..

[34] Ubeda C., Callejon R.M., Hidalgo C., Torija M.J., Mas A., Troncoso A.M., Morales M.L. (2011). Determination of major volatile compounds during the production of fruit vinegars by static headspace gas chromatography-mass spectrometry method. Food Res. Int..

[35] Yalçin O., Tekgündüz C., Öztürk M.M., Tekgündüz E. (2021). Investigation of the traditional organic vinegars by UV-VIS spectroscopy and rheology techniques. Spectrochim. Acta A.

[36] Codex Alimentarius Commission, 1987. Draft European Regional Standard for Vinegar. Rome, Italy. https://www.fao.org/fao-who-codexalimentarius/sh-proxy/en/?lnk=1&url=https%253A%252F%252Fworkspace.fao.org%252Fsites%252Fcodex%252FMeetings%252FCX-706-15%252Fal87_19e.pdf.

[37] Mohamad N.E., Yeap S.K., Lim K.L., Yusof H.M., Beh B.K., Tan S.W., Ho W.Y., Sharifuddin S.A., Jamaluddin A., Long K. (2015). Antioxidant effects of pineapple vinegar in reversing of paracetamol-induced liver damage in mice. Chin. Med..

[38] Roda A., Lucini L., Torchio F., Dordoni R., De Faveri D.M., Lambri M. (2017). Metabolite profiling and volatiles of pineapple wine and vinegar obtained from pineapple waste. Food Chem..

[39] Ren M., Wang X., Tian C., Li X., Zhang B., Song X., Zhang J. (2017). Characterization of organic acids and phenolic compounds of cereal vinegars and fruit vinegars in China. J. Food Process. Pres..

[40] Ordoudi S.A., Mantzouridou F., Daftsiou E., Malo C., Hatzidimitiou E., Nenadis N., Tsimidou M.Z. (2014). Pomegranate juice functional constituents after alcoholic and acetic acid fermentation. J. Funct. Food..

[41] Lin J.T., Liu S.C., Shen Y., Yang D.J. (2011). Comparison of various preparation methods for determination of organic acids in fruit vinegars with a simple ion-exclusion liquid chromatography. Food Anal. Method..

[42] Özen M., Özdemir N., Filiz B.E., Budak N.H., Kök-Taș T. (2020). Sour Cherry (*Prunus cerasus* L.) Vinegars produced from fresh fruit or juice concentrate: Bioactive compounds, volatile aroma compounds and antioxidant capacities. Food Chem..

[43] Ho C.W., Lazim A.M., Fazry S., Zaki U.K.H.H., Lim S.J. (2017). Effects of fermentation time and ph on soursop (*Annona muricata*) vinegar production towards its chemical compositions. Sains Malays..

[44] Coelho E., Geenisheva Z., Oliveira J.M., Teixeira J.A., Domingue L. (2017). Vinegar production from fruit concentrates: Effect on volatile composition and antioxidant activity. J. Food Sci. Technol. Mys..

[45] Kong C.T., Ho C.W., Alvin L.J.W., Iazim A., Fazry S., Lim S.J. (2018). Chemical changes and optimisation of acetous fermentation time and mother of vinegar concentration in the production of vinegar-like fermented papaya beverage. Sains Malays..

[46] Samad A., Azlan A., Ismail A. (2016). Therapeutic effects of vinegar: A review. Cur. Opin. Food Sci..

[47] Ozturk I., Caliskan O., Tornuk F., Ozcan N., Yalcin H., Baslar M., Sagdic O. (2015). Antioxidant, antimicrobial, mineral, volatile, physicochemical and microbiological characteristics of traditional home-made Turkish vinegars. LWT.

[48] Sengun I., Turp G.Y., Cicek S.N., Avci T., Ozturk B., Kilic G. (2021). Assessment of the effect of marination with organic fruit vinegars on safety and quality of beef. Int. J. Food Microbiol..

[49] Salanță L.C., Coldea T.E., Ignat M.V., Pop C.R., Tofană M., Mudura E., Borșa A., Pasqualone A., Zhao H. (2020). Non-alcoholic and craft beer production and challenges. Processes.

[50] Ducruet J., Rebenaque P., Diserens S., Kosinska-Cagnazzo A., Heritier I., Andlauer W. (2017). Amber ale beer enriched with goji berries—The effect on bioactive compound content and sensorial properties. Food Chem..

[51] Fanari M., Forteschi M., Sanna M., Piu P.P., Porcu M.C., D’hallewin G., Secchi N., Zinellu M., Pretti L. (2020). Pilot plant production of craft fruit beer using Ohmic-treated fruit puree. J. Food Process. Pres..

[52] Prasad M.P. (2014). In-vitro evaluation of antioxidant properties of fermented fruit beer samples. Int. J. Sci. Res..

[53] Martinez A., Vegara S., Marti N., Valero M., Saura D. (2017). Physicochemical characterization of special persimmon fruit beers using bohemian pilsner malt as a base. J. Inst. Brew..

[54] Nardini M., Garaguso I. (2020). Characterization of bioactive compounds and antioxidant activity of fruits beer. Food Chem..

[55] De Roos J., De Vuyst L. (2018). Microbial acidification, alcoholization, and aroma production during spontaneous lambic beer production. J. Sci. Food Agric..

[56] Witrick K., Pitts E.R., O’Keefe F.S. (2020). Analysis of Lambic beer volatiles during aging using gas chromatography-mass spectrometry (GCMS) and gas chromatography-olfactometry (GCO). Beverages.

[57] De Roos J., De Vuyst L. (2018). Acetic acid bacteria in fermented foods and beverages. Curr. Opin. Biotechnol..

[58] Verachtert H., Derdelinckx G. (2014). Belgian acidic beers, Daily reminiscences of the Past. Cerevisiae.

[59] Sugrue M., Dando R. (2018). Cross-modal influence of color from product and packaging alters perceived flavour of cider. J. Inst. Brew..

[60] Guiné R.P.F., Barroca M.J., Coldea T.E., Bartkiene E., Anjos O. (2021). Apple fermented products: An overview of technology properties and health effects. Processes.

[61] Venkatachalam K., Techakanon C., Thitithanakul S. (2018). Impact of the ripening stage of wax apples on chemical profiles of juice and cider. ACS Omega.

[62] AICV European Cider Trends 2020 Report. https://aicv.org/en/news/aicv-european-cider-trends-2020-now-published.

[63] Ye M., Yue T., Yuan Y. (2014). Evolution of polyphenols and organic acids during the fermentation of apple cider. J. Sci. Food Agric..

[64] Villar A., Vadillo J., Santos I., Gorritxategi E., Mabe J., Arnaiz A., Fernandez L.A. (2017). Cider fermentation process monitoring by Vis-Nir sensor system and chemometrics. Food Chem..

[65] Jarvis B., Batt C.A., Tortorello M.-L. (2014). Cider (Cyder; Hard Cider). Encyclopedia of Food Microbiology.

[66] Wei J., Zhang Y., Qiu Y., Guo H., Ju H., Wang Y., Yuan Y., Yue T. (2020). Chemical composition, sensorial properties, and aroma-active compounds of ciders fermented with *Hanseniaspora osmophila* and *Torulaspora quercuum* in co- and sequential fermentation. Food Chem..

[67] Magalhães F., Krogerus K., Vidgren V., Snadell M., Gibson B.R. (2017). Improved cider fermentation performance and quality with newly generated *Saccharomyces cerevisiae×Saccharomyces eubayanus* hybrids. J. Ind. Microbiol. Biotechnol..

[68] Akubor P. (2017). Characterization of fruit wines from baobab (*Adansonia digitata*), pineapple (*Ananas sativus*) and carrot (*Daucus carota*) tropical fruits. Asian J. Biotechnol. Bioresour. Technol..

[69] Swami S.B., Thakor N., Divate A.D. (2014). Fruit wine production: A review. J. Food Res. Techn..

[70] Klarić D.A., Klarići I., Mornar A., Velić N., Velić D. (2020). Assessment of bioactive phenolic compounds and antioxidant activity of blackberry wines. Foods.

[71] Wang C.-Y., Liu Y.W., Jia J.Q., Sivakumar T.R., Fan T., Gui Z.Z. (2013). Optimization of fermentation process for preparation of mulberry fruit wine by response surface methodology. Afr. J. Microbiol. Res..

[72] Chilaka C.A., Uchechukwu N., Obidiegwu J., Akpor O.B. (2010). Evaluation of the efficiency of yeast isolates from palmwine in diverse fruit wine production. Afr. J. Food Sci..

[73] Pino J., Oscar Q. (2011). Analysis of volatile compounds of mango wine. Food Chem..

[74] Ogodo A.C., Ugbogu O., Ugbogu E.A., Stephen E.C. (2015). Production of mixed fruit (pawpaw, banana and watermelon) wine using *Saccharomyces cerevisiae* isolated from palm wine. Springer Plus.

[75] Duarte W.F., Dias D.R., de Melo Pereira G.V., Gervasio I.M., Schwan R.F. (2009). Indigenous and inoculated yeast fermentation of gabiroba (*Campomanesia pubescens*) pulp for fruit wine production. J. Ind. Microbiol. Biotechnol..

[76] Jayabalan R., Waisundara V.Y., Grumezescu A.M., Holban A.M. (2019). 12—Kombucha as a Functional Beverage.

[77] Villarreal-Soto S.A., Beaufort S., Bouajila J., Souchard J.-P., Taillandier P. (2018). Understanding Kombucha tea fermentation: A review. J. Food Sci..

[78] Zubaidah E., Dewantari F.J., Novitasari F.R., Srianta I., Blanc P.J. (2018). Potential of snake fruit (*Salacca zalacca* (Gaerth.) Voss) for the development of a beverage through fermentation with the Kombucha consortium. Biocatal. Agric..

[79] Sharifudin S.A., Ho W.Y., Yeap S.K., Abdullah R., Koh S.P. (2021). Fermentation and characterisation of potential kombucha cultures on papaya-based substrates. LWT.

[80] Akbarirad H., Assadi M.M., Pourahmad R., Khaneghah A.M. (2017). Employing of the different fruit juices substrates in vinegar Kombucha preparation. Curr. Nut. Food Sci..

[81] Rahmani R., Beaufort S., Villarreal-Soto S.A., Taillandier P., Bouajila J., Debouba M. (2019). Kombucha fermentation of African mustard (*Brassica tournefortii*) leaves: Chemical composition and bioactivity. Food Biosci..

[82] Vázquez-Cabral B.D., Rocha-Guzmán N.E., Gallegos-Infante J.A., González-Herrera S.M., González-Laredo R.F., Moreno-Jiménez M.R., Córdova-Moreno I.T.S. (2014). Chemical and sensory evaluation of a functional beverage obtained from infusions of oak leaves (*Quercus resinosa*) inoculated with the Kombucha consortium under different processing conditions. Nutrafoods.

[83] Zofia N.-Ł., Aleksandra Z., Tomasz B., Martyna Z.-D., Magdalena Z., Zofia H.-B., Tomasz W. (2020). Effect of Fermentation Time on Antioxidant and Anti-Ageing Properties of Green Coffee Kombucha Ferments. Molecules.

[84] Watawana M., Jayawardena N., Waisundara V. (2015). Enhancement of the functional properties of coffee through fermentation by “tea fungus” ( Kombucha). J. Food Process. Preserv..

[85] Bueno F., Chouljenko A., Sathivel S. (2021). Development of coffee kombucha containing *Lactobacillus rhamnosus* and *Lactobacillus casei*: Gastrointestinal simulations and DNA microbial analysis. LWT.

[86] Aspiyanto S., Iskandar J.M., Melanie H., Maryati Y., Lotulung P.D. Characteristic of fermented spinach (*Amaranthus* spp.) polyphenol by kombucha culture for antioxidant compound. Proceedings of the International Symposium on Applied Chemistry (ISAC).

[87] Malbaša R., Vitas J., Lončar E., Kravić S. (2011). Influence of fermentation temperature on the content of fatty acids in low energy milk-based kombucha products. Acta Period. Technol..

[88] Malbaša R., Lončar E., Djurić M. (2008). Comparison of the products of Kombucha fermentation on sucrose and molasses. Food Chem..

[89] Kabak B., Dobson A.D.W. (2011). An introduction to the traditional fermented foods and beverages of Turkey. Crit. Rev. Food Sci. Nutr..

[90] Misihairabgwi J., Cheikhyoussef A. (2017). Traditional fermented foods and beverages of Namibia. J. Ethn. Foods.

[91] Perez-Armendariz B., Cardoso-Ugarte G.A. (2020). Traditional fermented beverages in Mexico: Biotechnological, nutritional, and functional approaches. Int. Food Res. J..

[92] Motlhanka K., Zhou N., Lebani K. (2018). Microbial and chemical diversity of traditional non-cereal based alcoholic beverages of Sub-Saharan Africa. Beverages.

[93] Patel A.R. (2017). Probiotic fruit and vegetable juices-recent advances and future perspective. Int. Food Res. J..

[94] Sun-Waterhouse D. (2011). The development of fruit-based functional foods targeting the health and wellness market: A review. Int. J. Food Sci. Technol..

[95] Russel W., Duthie G. (2011). Plant secondary metabolites and gut health: The case for phenolic acids. Proc. Nutr. Soc..

[96] Štochmal’ova A., Sirotkin A., Kadasi A., Alexa R. (2013). Physiological and medical effects of plant flavonoid quercetin. J. Microbiol. Biotechnol. Food Sci..

[97] Das A.M., Goud V.V., Das C., Grumuzescu A.M., Holban A.M. (2019). Phenolic compounds as functional ingredients in beverages. Value-Added Ingredients and Enrichments of Beverages.

[98] Ji-Yong S., Xiao-Bo Z., Xiao-Wei H., Jie-Wen Z., Yanxiao L., Limin H., Jianchun Z. (2013). Rapid detecting total acid content and classifying different types of vinegar based on near infrared spectroscopy and least-squares support vector machine. Food Chem..

[99] Choudhary A., Kumar V., Kumar S., Majid I., Aggarwal P., Suri S. (2020). 5-Hydroxymethylfurfural (HMF) formation, occurrence and potential health concerns: Recent developments. Toxin Rev..

[100] Theobald A., Müller A., Anklam E. (1998). Determination of 5-hydroxymethylfurfural in vinegar sam-ples by HPLC. J. Agr. Food Chem..

[101] Bounihi A., Bitam A., Bouazza A., Yargui L., Koceir E.A. (2017). Fruit vinegars attenuate cardiac injury via anti inflammatory and antiadiposity action in high-fat diet-induced obese rats. Pharm. Biol..

[102] Bouazza A., Bitam A., Amiali M., Bounihi A., Yargui L., Koceir E.A. (2016). Effect of fruit vinegar on liver damage and oxidative stress in high-fat-fed rats. Pharm. Biol.

[103] Shahidi F., McDonald J., Chandrasekara A., Zhong Y. (2008). Phytochemicals of foods, beverages and fruit vinegars: Chemistry and health effects. Asia Pac. J. Clin. Nutr..

[104] Danielewski M., Matuszewska A., Nowak B., Kucharska A.Z., Sozanski T. (2020). The Effects of natural iridoids and anthocyanins on selected parameters of liver and cardiovascular system functions. Oxid. Med. Cell. Longev..

[105] Kawa-Rygielska J., Adamenko K., Kucharska A.Z., Prorok P., Piorecki N. (2019). Physicochemical and antioxidative properties of Cornelian cherry beer. Food Chem..

[106] Septembre-Malaterre A., Remize F., Poucheret P. (2018). Fruits and vegetables, as a source of nutritional compounds and phytochemical: Changes in bioactive compounds during lactic fermentation. Food Res. Int..

[107] LeBlanc J.G., Milani C., Savoy de Giori G., Sesma F., van Sinderen D., Ventura M. (2013). Bacteria as vitamin suppliers to their host: A gut microbiota perspective. Curr. Opin. Biotechnol..

[108] Santos F., Wegkamp A., De Vos W., Smid E., Hugenholyz J. (2018). High-level folate production in fermented foods by the B_12_ producer *Lactobacillus reuteri* JCM1112. Appl. Environ. Microbiol..

[109] Giri S.S., Sukumaran S., Sen S.S., Park S.C. (2018). Use of a potential probiotic, *Lactobacillus casei* L4, in the Preparation of fermented coconut water beverage. Front. Microbiol..

[110] Klopotek Y., Otto K., Böhm V. (2005). Processing strawberries to different products alters contents of vitamin C, total phenolics, total anthocyanins, an antioxidant capacity. J. Agr. Food Chem..

[111] AdebayoTayo B., Akpeji S. (2016). Probiotic viability, physicochemical and sensory properties of probiotic pineapple juice. Fermentation.

[112] Kwaw E., Ma Y., Tchabo W., Apaliya M.T., Sackey A.S., Wu M., Xiao L. (2018). Impact of ultrasonication and pulsed light treatments on phenolics concentration and antioxidant activities of lactic-acid-fermented mulberry juice. LWT.

[113] Bortolini D.G., Benvenutti L., Demiate I.M., Nogueira A., Alberti A., Zielinski A.A.F. (2020). A new approach to the use of apple pomace in cider making for the recovery of phenolic compounds. LWT.

[114] Benvenutti L., Bortolini D.G., Nogueira A., Zielinski A.A.F., Alberti A. (2019). Effect of addition of phenolic compounds recovered from apple pomace over cider quality. LWT.

[115] Callemien D., Collin S. (2009). Structure, Organoleptic Properties, quantification methods, and stability of phenolic compounds in beer-a review. Food Rev. Int..

[116] Li Z.C., Chi L.Z., Zhu J.K., Zhang Y.Y., Wang Q.J., He P.G., Fang Y.Z. (2011). Simultaneous determination of active ingredients in blueberry wine by CE-AD. Chin. Chem. Lett..

[117] Fan L., Wang Y., Xie P., Zhang L., Li Y., Zhou J. (2019). Copigmentation effects of phenolics on color enhancement and stability of blackberry wine residue anthocyanins: Chromaticity, kinetics and structural simulation. Food Chem..

[118] Mudnic I., Budimir D., Modun D., Gunjaca G., Mekinić I.G., Sskroza D., Katalinic V., Ljubenkov I., Boban M. (2012). Antioxidant and vasodilatory effects of blackberry and grape wines. J. Med. Food.

[119] Dini I., Grumezescu A.M., Holban A.M. (2019). An overview of functional beverages. Functional and Medicinal Beverages.

[120] Tamang J.P., Shin D., Jing S.-J., Chae S.-E. (2016). Functional properties of microorganisms in fermented foods. Front. Microbiol..

[121] Perricone M., Bevilacqua A., Altieri C., Sinigaglia M., Corbo M.R. (2015). Challenges for the production of probiotic fruit juices. Beverages.

[122] Nazhand A., Souto E.B., Lucarini M., Souto S.B., Durazzo A., Santini A. (2020). Ready to use therapeutical beverages: Focus on functional beverages containing probiotics, prebiotics and synbiotics. Beverages.

[123] Raman M., Ambalam P., Doble M., Grumezescu A.M., Holban A.M. (2019). 9-Probiotics, prebiotics, and fibers nutritive and functional beverages. Nutrients in Beverages.

[124] Di Cagno R., Filannino P., Cantatore V., Polo A., Celano G., Martinovic A., Cavoski I., Gobbetii M. (2020). Design of potential probiotic yeast starters tailored for making a cornelian cherry (*Cornus mas* L.) functional beverage. Int. J. Food Microbiol..

[125] Valero-Cases E., Cerda-Bernad D., Pastor J.J., Frutos M.J. (2020). Non-dairy fermented beverages as potential carriers to ensure probiotics, prebiotics, and bioactive compounds arrival to the gut and their health benefits. Nutrients.

[126] Yang X., Zhou J., Fan L., Zhen Q., Chen Q., Zhao L. (2018). Antioxidant properties of a vegetable-fruit beverage fermented with two *Lactobacillus plantarum* series. Food Sci. Biotechnol..

[127] Güney D., Güngörmüşler M. (2021). Development and Comparative Evaluation of a Novel Fermented Juice Mixture with Probiotic Strains of Lactic Acid Bacteria and Bifidobacteria. Prebiotics Antimicrob. Proteins.

[128] Lamsal B.P. (2012). Production, health aspects and potential food uses of dairy prebiotic galactooligosaccharides. J. Sci. Food Agric..

[129] Saad N., Delattre C., Urdaci M.C., Schmitter J.M., Bressollier P. (2013). An overview of the last advances in probiotic and prebiotic field. LWT.

[130] Fonteles T.V., Rodrigues S. (2018). Prebiotic in fruit juice: Processing challanges, advances, and perspective. Curr. Opin. Food Sci..

[131] Corbo M.R., Bevilacqua A., Petruzzi L., Casanova F.P., Sinigaglia M. (2014). Functional Beverages: The emerging side of functional foods. Compr. Rev. Food Sci. Food Saf..

[132] Costabile A., Walton G.E., Tzoetzis G., Vulevic J., Charalampopoulos D., Gibson G.R. (2015). Effects of orange juice formulation on prebiotic functionality using an in vitro colonic model system. PLoS ONE.

[133] Filannino P., Azzi L., Cavoski I., Vincentini O., Rizzello C.G., Gobbetti M., Di Cagno R. (2013). Exploitation of the health-promoting and sensory properties of organic pomegranate (*Punica granatum* L.) juice through lactic acid fermentation. Int. J. Food Microbiol..

[134] Murphy M.M., Barrai L.M., Herman D., Bi X., Cheatham R., Randolph R.K. (2012). Phytonutrient intake by adults in the USA in relation to fruit and vegetable consumption. J. Acad. Nutr. Diet..

[135] Negi B., Kaur R., Dey G. (2013). Protective effects of a novel sea buckthorn wine on oxidative stress and hypercholesterolemia. Food Funct..

[136] Gallie D.R. (2013). L-ascorbic acid: A multifunctional molecule supporting plant growth and development. Scientifica.

[137] Brownawell A., Caers W., Gibson G.R., Kendall C.W.C., Lewis K.D., Ringel Y., Slavin J.L. (2012). Prebiotics and the health benefits of fiber:current regulatory status, future research, and goals. J. Nutr..

[138] White J., Hekmat S. (2018). Development of probiotic fruit juices using *Lactobacillus rhamnosus* GR-1 Fortified with short chain and long chain inulin fiber. Fermentation.

[139] Ghafari S., Ansari S. (2018). Microbial viability, physico-chemical properties and sensory evaluation of pineapple juice enriched with Lactobacillus casei, Lactobacillus rhamnosus and inulin during refrigerated storage. J. Food Meas. Charact..

[140] Khezri S., Mahmoudi R., Dehghan P. (2018). Fig juice fortified with inulin and *Lactobacillus delbrueckii*: A promising functional food. Appl. Food Biotechnol..

[141] Blanco-Morales V., Lopez-Garcia G., Cilla A., Garcia-Llatas G., Barbera R., Lagarda M.J., Sanchez-Siles L.M., Alegria A. (2018). The impact of galactooligosaccharides on the bioaccessibility of sterols in a plant sterol-enriched beverage: Adaptation of the harmonized INFOGEST digestion method. Food Funct..

[142] Lopez-Garcia G., Cilla A., Barberá R., Martinez S.G., Martorell P., Alegria A. (2020). Effect of plant sterol and galactooligosaccharides enriched beverages on oxidative stress and longevity in *Caenorhabditis elegans*. J. Funct. Foods.

[143] Ghavidel R.A., Karimi M., Davoodi M., Jahanbani R., Asl A.F.A. (2014). Effect of fructooligosaccharide fortification on quality characteristic of some fruit juice beverages (apple & orange juice). Intl. J. Farm. Alli. Sci..

[144] Almeida F.D.L., Gomes W.F., Cavalcante R.S., Tiwari B.K., Cullen P.J., Frias J.M., Bourke P., Fernandes F.A.N., Rodrigues S. (2017). Fructooligosaccharides integrity after atmospheric cold plasma and high-pressure processing of a functional orange juice. Food Res. Int..

[145] Di Cagno R., Mazzacane F., Rizzello C.G., De Angelis M., Giuliani G., Meloni M., De Servi M., Gobbetti M. (2010). Synthesis of gamma-aminoburyric acid (GABA) by *Lactobacillus plantarum* DSM19463: Functional grape must beverage and dermatological applications. Appl. Microbiol. Biotechnol..

[146] de Sá L.Z.M., Castro P.F., Lino F.M., Bernardes M.J., Viegas J.C., Dinis T.C., Santana M.J., Romao W., Vaz B.G., Lião L.M. (2014). Antioxidant potential and vasodilatory activity of fermented beverages of jabuticaba berry (*Myrciaria jaboticaba*). J. Funct. Foods.

[147] Mantzourani I., Terpou A., Alexopoulos A., Bezirtzoglou E., Bekatorou A., Plessas S. (2019). Production of a potentially synbiotic fermented *Cornelian cherry* (*Cornus mas* L.) beverage using *Lactobacillus paracasei* K5 immobilized on wheat bran. Biocatal. Agric. Biotechnol..

[148] Yonekura S., Okamoto Y., Okawa T., Hisamitsu M., Chazono H., Kobayashi K., Sakurai D., Horiguchi S., Hanazawa T. (2009). Effects of daily intake of *Lactobacillus paracasei* strain KW3110 on Japanese cedar pollinosis. Allergy Asthma Proc..

[149] Hütt P., Koll P., Stsepetova J., Alvarez B., Mändar R., Andersen K.K., Marcotte H., Hammarström L., Mikelsaar M. (2011). Safety and persistence of orally administered human Lactobacillus sp. strains in healthy adults. Benef. Microbes.

[150] Antolak H., Piechota D., Kucharska A. (2021). Kombucha tea—A double power of bioactive compounds from tea and symbiotic culture of bacteria and yeasts (SCOBY). Antioxidants.

[151] Rodino S., Butu M., Grumuzescu A.M., Holban A.M. (2018). 3-Herbal extracts-new trends in functional and medicine beverages. Functional and Medicine Beverages.

[152] Azmir J., Zaidul I.S.M., Rahman M.M., Sharif K.M., Mohamed A., Sahena F., Jahurul M.H.A., Ghafoor K., Norulaini N.A.N., Omar A.K.M. (2013). Techniques for extraction of bioactive compounds from plant materials: A review. J. Food Eng..

[153] Tonutti I., Liddle P. (2010). Aromatic plants in alcoholic beverages. A review. Flavour Fragr. J..

[154] Gonzalez-Manzano S., Rivas-Gonzalo J.C., Santos-Buelge C. (2004). Extraction of flavan-3-ols from grape seed and skin into wine using simulated maceration. Anal. Chim. Acta..

[155] Lou X., Guo X., Wang K., Wu C., Jin Y., Lin Y., Xu H., Habba M., Yuan L. (2021). Phenolic profiles and antioxidant activity of *Crataegus pinnatifida* fruit infusion and decoction and influence of in vitro gastrointestinal digestion on their digestive recovery. LWT.

[156] Maran J.P., Priya B., Manikandan S. (2014). Modeling and optimization of supercritical fluid extraction of anthocyanin and phenolic compounds from *Syzygium cumini* fruit pulp. J. Food Sci. Technol..

[157] Teng W.-H., Chen C.C., Chung R.-S. (2005). HPLC comparison of supercritical fluid extraction and solvent extraction of coumarins from the peel of *Citrus maxima* fruit. Phytochem. Anal..

[158] Ruiz-Rodríguez A., Fornari T., Jaime L., Vázquez E., Amador B., Nieto J.A., Yuste M., Mercader M., Reglero G. (2012). Supercritical CO2 extraction applied toward the production of a functional beverage from wine. J. Supercrit. Fluid..

[159] Silva E.K., Bargas M.A., Arruda H.S., Vardanega R., Pastore G.M., Meireles M.A.A. (2020). Supercritical CO2 processing of a functional beverage containing apple juice and aqueous extract of *Pfaffia glomerata* roots: Fructooligosaccharides chemical stability after non-thermal and thermal treatments. Molecules.

[160] Teng H., Lee W.Y., Choi Y.H. (2013). Optimization of microwave-assisted extraction for anthocyanins, polyphenols, and antioxidants from raspberry (*Rubus Coreanus* Miq.) using response surface methodology. J. Sep. Sci..

[161] Singh R., Kumar M., Mittal A., Mehta P.K. (2016). Microbial enzymes: Industrial progress in 21st century. 3 Biotechnol..

[162] Sattar S., Imran M., Mushtaq Z., Ahmad M.H., Arshad M.S., Holmes M., Maycock J., Nisar M.F., Khan M.K. (2020). Retention and stability of bioctive compounds in functional peaches beverage using pasteurization, microwave and ultrasound technologies. Food Sci. Biotechnol..

[163] Reddy V.B., Moniruzzaman M., Madhavi V., Jaafar J., Atta-ur-Rahman (2020). Chapter 8-Recent improvements in the extraction, cleanup and quantification of bioactive flavonoids. Studies in Natural Products Chemistry.

[164] Khandpur P., Gogate P.R. (2015). Effect of novel ultrasound based processing on the nutrition quality of different fruit and vegetable juices. Ultrason. Sonochem..

[165] Jiang X., Lu Y., Liu S.Q. (2020). Effects of pectinase treatment on the physicochemical and oenological properties of red dragon fruit wine fermented with *Torulaspora delbrueckii*. LWT.

[166] Dinkova R., Heffels P., Shikov V., Weber F., Schieber A., Mihalev K. (2014). Effect of enzyme-assisted extraction on the chilled storage stability of bilberry (*Vaccinium myrtillus* L.) anthocyanins in skin extracts and freshly pressed juice. Food Res. Int..

[167] Kim B.H.M., Park S.K. (2017). Enhancement of volatile aromatic compounds in black raspberry wines via enzymatic treatment: Volatiles in black raspberry wine via enzymatic treatment. J. Inst. Brew..

[168] Yang N., Huang K., Lyu C., Wang J. (2016). Pulsed electric field technology in the manufacturing processes of wine, beer, and rice wine: A review. Food Control.

[169] Al Daccache M., Koubaa M., Salameh D., Vorobiev E., Maroun R.G., Louka N. (2020). Control of the sugar/ethanol conversion rate during moderate pulsed electric field-assisted fermentation of *Hanseniaspora* sp. strain to produce low-alcohol cider. Innov. Food Sci. Emerg..

[170] Mota M.J., Lopes R.P., Koubaa M., Roohinejad S., Barba F.J., Delgadilo I., Saraiva J.A. (2017). Fermentation at non-conventional conditions in food- and bio-sciences by the application of advanced processing technologies. Crit. Rev. Biotechnol..

[171] Siddeeg A., Zeng X.-A., Rahaman A., Manzoor M.F., Ahmed Z., Ammar A.-F. (2019). Effect of pulsed electric field pretreatment of date palm fruits on free amino acids, bioactive components, and physicochemical characteristics of the alcoholic beverage. J. Food Sci..

[172] Delsart C., Cholet C., Ghidossi R., Grimi N., Gontier E., Geny L., Vorobiev E., Mietton-Peuchot M. (2013). Effects of pulsed electric fields on Cabernet Sauvignon grape berries and on the characteristics of wines. Food Bioproc. Technol..

[173] Gabrić D., Barba F., Roohinejad S., Gharibzahedi S.M.T., Radojčin M., Putnik P., Kovačević D.B. (2018). Pulsed electric fields as an alternative to thermal processing for preservation of nutritive and physicochemical properties of beverages: A review. J. Food Process. Eng..

[174] Yi J., Kebede B.T., Kristiani K., Grauwet T., van Loey A., Hendrickx M. (2017). Minimizing quality changes of cloudy apple juice: The use of kiwifruit puree and high pressure homogenization. Food Chem..

[175] Morata A., Loira I., Vejarano R., Bañuelos M.A., Sanz P.D., Otero L., Suarez-Lepe J.A. (2014). Grape processing by high hydrostatic pressure: Effect on microbial populations, phenol extraction and wine quality. Food Bioproc. Technol..

[176] Garcia C., Guerin M., Souidi K., Remize F. (2020). Lactic fermented fruit or vegetable juices: Past, present and future. Beverages.

[177] Jincy G.M., Rastogi N.K. (2016). High pressure procesing for food fermentation. Novel Food Fermentation Technologies.

[178] Maza M.A., Martinez J.M., Hernandez-Orte P., Cebrian G., Sanchez-Gimeno A.C., Alvarez I., Raso J. (2019). Influence of pulsed electric fields on aroma and polyphenolic compounds of Garnacha wine. Food Bioprod. Process..

[179] Xu L.-F., Tang Z.-S., Wen Q.-H., Zeng X.-A., Brennan C., Niu D. (2019). Effects of pulsed electric fields pretreatment on the quality of jujube wine. Int. J. Food Sci. Technol..

[180] Kaur M., Sahota P., Sharma N., Kaur K., Sood B. (2018). Enzymatic production of debittered Kinnow juice and beverage. J. Curr. Microbiol. Appl. Sci..

[181] Guo J., Yan Y., Wang M., Wu Y., Liu S.-Q., Chen D., Lu Y. (2018). Effects of enzymatic hydrolysis on the chemical constituents in jujube alcoholic beverage fermented with *Torulaspora delbrueckii*. LWT.

[182] Toy J.Y.H., Lu Y., Huang D., Matsumura K., Liu S.Q. (2020). Enzymatic treatment, unfermented and fermented fruit-based products: Current state of knowledge. Crit. Rev. Food. Sci. Nutr..

[183] Belda I., Conchilo L.B., Ruiz J., Navascues E., Marquina D., Santos A. (2016). Selection and use of pectinolytic yeasts for improving clarification and phenolic extraction in winemaking. Int. J. Food Microbiol..

[184] Ferreira Rosas L., Alves Macedo J., Lima Ribeiro M., Alves Macedo G. (2013). Improving the chemopreventive potential of orange juice by enzymatic biotransformation. Food Res. Int..

[185] Liu S., Chang X., Liu X., Shen Z. (2016). Effects of pretreatments on anthocyanin composition, phenolics contents and antioxidant capacities during fermentation of hawthorn (*Crataegus pinnatifida*) drink. Food Chem..

[186] Kubo M.T.K., Curet S., Augusto P.E.D., Boillereaux L. (2019). Multiphysics modeling of microwave processing for enzyme inactivation in fruit juices. J. Food Technol..

[187] Mendes-Oliveira G., Deering A.J., San Martin-Gonzalez F.M., Campanella O.H. (2020). Microwave pasteurization of apple juice: Modeling the inactivation of *Escherichia coli* O157:H7 and *Salmonella Typhimurium* at 80–90°C. Food Microbiol..

[188] Salazar-Gonzalez C., San Martin-Gonzalez F.M., Lopez-Malo A., Sosa-Morales M.E. (2012). Recent studies related to microwave processing of fluid foods. Food Bioproc. Technol..

[189] Swamy G.J., Muthukumarappan K., Asokapandian S. (2018). Chapter 23—Ultrasound for fruit juice preservation; fruit juices- extraction, composition. Quality and Analysis.

[190] Zhai X., Wang X., Wang X., Zhang H., Ji Y., Ren D., Lu J. (2021). An efficient method using ultrasound to accelerate aging in crabapple (*Malus asiatica*) vinegar produced from fresh fruit and its influencing mechanism investigation. Ultrason. Sonochem..

[191] Wang J., Xie B., Sun Z. (2020). Quality parameters and bioactive compound bioaccessibility changes in probiotics fermented mango juice using ultraviolet-assisted ultrasonic pre-treatment during cold storage. LWT.

[192] Paniwnyk L. (2017). Applications of ultrasound in processing of liquid foods: A review. Ultrason. Sonochem..

[193] Costa M.G., Fonteles T.D., Tiberio de Jesus A.L., Rodrigues S. (2013). Sonicated pineapple juice as substrate for *L. casei* cultivation for probiotic beverage development: Process optimisation and product stability. Food Chem..

[194] Guimarães J.T., Silva E.K., Ranadheera S., Moraes J., Raices R., Silva M.C., Ferreira M.S., Freitas M.Q., Meireles M.A.A., Cruz A.G. (2019). Effect of high-intensity ultrasound on the nutritional profile and volatile compounds of a prebiotic soursop whey beverage. Ultrason. Sonochem..

[195] Zhou L., Guan Y., Bi J., Liu X., Yi J., Chen Q., Wu X., Zhou M. (2017). Change of the rheological properties of mango juice by high pressure homogenization. LWT.

[196] Bansal V., Jabeen K., Rao P.S., Prasad P., Yadav S.K. (2018). Effect of high pressure processing (HPP) on microbial safety, physicochemical properties, and bioactive compounds of whay-based sweet lime (whey-lime) beverage. J. Food Meas. Charact..

[197] Nayak P.K., Rayaguru K., Krishnan R.K. (2016). Quality comparison of elephant apple juices after high-pressure processing and thermal treatment. J. Sci. Food Agric..

[198] Morata A., Guamis B. (2020). Use of UHPH to obtain juices with better nutritional quality and healthier wines with low levels of SO_2_. Front. Nutr..

[199] Huang H.-W., Wu S., Lu J.K., Shyu Y.T., Wang C.Y. (2017). Current status and future trends of high-pressure processing in food industry. Food Control.

[200] Balasubramaniam V.M., Martinez-Monteagudo S.I., Gupta R. (2015). Principles and application of high pressure-based technologies in the food industry. Annu. Rev. Food Sci. Technol..

[201] Ciani M., Canonico L., Oro L., Comitini F., Grumuzescu A.M., Holban A.M. (2020). 14-Footprint of nonconventional yeasts and their contribution in alcoholic fermentation. Biotechnological Progress and Beverage Consumption.

[202] Benito S., Palomero F., Morata A., Calderon F., Suarez-Lepe J.A. (2013). New applications for *Schizosaccharomyces pombe* in the alcoholic fermentation of red wines. Int. J. Food Sci. Technol..

[203] Mas A., Guillamon J.M., Torija M.J., Beltran G., Cerezo A.B., Troncoso A.M., Garcia-Parrilla C. (2014). Bioactive compounds derived from the yeast metabolism of aromatic amino acids during alcoholic fermentation. BioMed Res. Int..

[204] Rodriguez-Naranjo I., Gil-Izquierdo A., Troncoso A.M., Cantos-Villar E., Garcia-Parrilla C. (2011). Melatonin is synthesised by yeast during alcoholic fermentation in wines. Food Chem..

[205] Fernández-Pachón M.-S., Medina S., Herrero-Martin G., Cerrillo I., Berna G., Escudero-Lopez B., Ferreres F., Martin F., Garcia-Parrilla M.C., Gil-Izquierdo A. (2014). Alcoholic fermentation induces melatonin synthesis in orange juice. J. Pineal Res..

[206] Feng X., Wang M., Zhao Y., Han P., Dai Y. (2014). Melatonin from different fruit sources, functional roles, and analytical methods. Trends Food Sci. Technol..

[207] Cerrillo I., Escudero-Lopez B., Hornero-Mendez D., Martin F., Fernandez-Pachon M.-S. (2014). Effect of alcoholic fermentation on the carotenoid composition and provitamin a content of orange juice. J. Agric. Food Chem..

[208] Escudero-Lopez B., Cerrillo I., Herrero-Martin G., Hornero-Mendez D., Gil-Izquierdo A., Medina S., Ferreres F., Berna G., Martin F., Fernandez-Pachon M.-S. (2012). Fermented orange juice: Source of higher carotenoid and flavanone contents. J. Agric. Food Chem..

[209] Sengun I.Y. (2017). Acetic Acid Bacteria. Fundamentals Food Applications.

[210] Yamada Y., Matsushita K., Toyama H., Tonouchi N., Okamoto-Kainuma A. (2016). Systematics of Acetic Acid Bacteria. Acetic Acid Bacteria Ecology and Physiology.

[211] Gomes R.J., de Fatima B.M., de Freitas R.M., Castro-Gomez R.J.H., Spinosa W.A. (2018). Acetic acid bacteria in the food industry: Systematics, characteristics and applications. Food Technol. Biotechnol..

[212] Giuffré A.M., Zappia C., Capocasale M., Poiana M., Sidari R., Di Donna L., Bartella L., Sindona G., Corradini G., Giudici P. (2018). Vinegar production to valorise *Citrus bergamia* by-products. Eur. Food Res. Technol..

[213] Malo P., Urquhart E.A., Carl Batt C., Pradip P. (2016). Fermented foods: Use of starter cultures. Encyclopedia of Food and Health.

[214] Muñoz R., Moreno-Arribas V.M., de las Rivas B., Santiago A.C., Munoz R., Gonzalez Garcia R. (2011). Chapter 8-Lactic Acid Bacteria. Molecular Wine Microbiology.

[215] Muhialdin B.J., Kadum H., Zarei M., Hussin A.S.M. (2020). Effects of metabolite changes during lacto-fermentation on the biological activity and consumer acceptability for dragon fruit juice. LWT.

[216] Muhialdin B.J., Kadum H., Hussin A.S.M. (2021). Metabolomics profiling of fermented cantaloupe juice and the potential application to extend the shelf life of cantaloupe juice for six months at 8 °C. Food Control.

[217] Ceci C., Lacal P.M., Tentori L., De Martino M.G., Miano R., Graziani G. (2018). Experimental evidence of the antitumor, antimetastatic and antiangiogenic activity of ellagic acid. Nutrients.

[218] Chen R., Chen W., Chen H., Zhang G., Chen W. (2018). Comparative evaluation of the antioxidant capacities, organic acids, and volatiles of papaya juices fermented by *Lactobacillus acidophilus* and *Lactobacillus plantarum*. J. Food Qual..

[219] Furukawa S., Watanabe T., Toyama H., Morinaga Y. (2013). Significance of microbial symbiotic coexistence in traditional fermentation. J. Biosci. Bioeng..

[220] Stadie J., Gulitz A., Ehrmann M., Vogel R. (2013). Metabolic activity and symbiotic interactions of lactic acid bacteria and yeasts isolated from water kefir. Food Microbiol..

[221] Sabokbar N., Khodaiyan F. (2015). Characterization of pomegranate juice and whey based novel beverage fermented by kefir grains. J. Food Sci. Technol..

[222] Sabokbar N., Moosabi-Nasab M., Khodaiyan F. (2015). Preparation and characterization of an apple juice and whey based novel beverage fermented using kefir grains. Food Sci. Biotechnol..

[223] Kazakos S., Mantzourani I., Nouska C., Alexopoulos A., Bezirtzoglou E., Bekatorou A., Plessas S., Varzakas T.S. (2016). Production of low-alcohol fruit beverages through fermentation of pomegranate and orange juices with kefir grains. Curr. Res. Nutr. Food Sci..

[224] Bellut K., Krogerus K., Arendt E.K. (2020). *Lachancea fermentati* strains isolated from Kombucha: Fundamental insights, and practical application in low alcohol beer brewing. Front. Microbiol..

[225] Mohanty D., Misra S., Mohapatra S., Sahu P.S. (2018). Prebiotics and synbiotics: Recent concepts in nutrition. Food Biosci..

[226] Trigo J.P., Alexandre E.M.C., Saraiva J.M.A., Pintado M.E. (2019). High value-added compounds from fruit and vegetable by-products –characterization, bioactivities, and application in the development of novel food products. Crit. Rev. Food Sci. Nutr..

[227] Ferrentino G., Asaduzzaman M.D., Scampicchio M.M. (2018). Current technologies and new insights for the recovery of high valuable compounds from fruits by-products. Crit. Rev. Food Sci. Nutr..

[228] Campos D.A., Gomez-Garcia R., Vilas-Boas A.A., Madureira A.R., Pintado M.M. (2020). Management of fruit industrial by-products-a case study on circular economy approach. Molecules.

